# Hydrogel-Mediated Delivery of Drug Nanocarriers in the Oedematous Postoperative Brain

**DOI:** 10.1007/s11095-026-04155-8

**Published:** 2026-07-14

**Authors:** Noel Benton, Wenbo Zhan

**Affiliations:** School of Engineering, University of Aberdeen, Aberdeen, AB24 3UE UK

**Keywords:** cancer recurrence, drug transport, hydrogel, mechanistic modelling, nanocarrier

## Abstract

**Purpose and Objectives:**

Hydrogel-nanocarrier systems have emerged as a clinically implementable strategy to reduce glioblastoma recurrence following surgery. Despite demonstrated feasibility, the influence of intrinsic tissue and nanocarrier properties on delivery outcomes remains insufficiently understood. This study aims to quantify their individual effects on delivery performance.

**Methods:**

A mathematical model is applied to simulate hydrogel-mediated nanocarrier delivery to the oedematous postoperative brain. Simulations are conducted in a realistic 3-D brain geometry rebuilt from medical imaging data. Nine factors are investigated, including drug release rate, nanocarrier diffusivity, blood drainage rate, partitioning at the cell membrane and within cells, drug loading dose, oedema severity, blood pressure, and tissue permeability.

**Results:**

The modelling reveals distinct responses of delivery outcomes to each factor. Optimal ranges are identified that maximise drug availability and/or improve distribution volume and uniformity. The results suggest that drug delivery is controlled predominantly by drug release and elimination rather than transport.

**Conclusions:**

These findings enhance understanding of the coupled interactions between drug transport and brain tissue in the post-operative environment and provide a quantitative reference for the optimisation of hydrogel-nanocarrier delivery systems to improve inhibition of glioblastoma recurrence.

## Introduction

Glioblastoma is the most common and aggressive primary brain malignancy in adults [1]. It is characterised by rapid progression and a lack of specific early-stage symptoms, potentially leading to late diagnosis. The current standard of care comprises maximal surgical resection followed by radiotherapy and chemotherapy [2], which extends median survival from approximately 6 to 15 months [3]. Despite this improvement, the prognosis remains poor. More than 90% of tumours recur within 12 months, primarily due to residual infiltrative cancer cells that evade resection and persist in the peritumoural region [4–6]. Clinical observations indicate that recurrence predominantly occurs within 2 cm of the resection margin [7]. Consequently, the 5-year survival rate remains below 5% [8], underscoring the urgent need for localised therapeutic strategies capable of targeting residual disease and preventing recurrence.

Hydrogels have emerged as promising local drug delivery platforms to address this challenge [9, 10]. Following tumour resection, drug-loaded hydrogels can be implanted into the surgical cavity, where their fluidic nature enables conformal contact with the highly irregular cavity surface. These systems provide sustained release of anticancer agents, thereby maintaining therapeutic concentrations in the surrounding tissue. More recently, nanocarrier-encapsulated drugs have been incorporated into hydrogels to modulate release kinetics, reduce premature clearance, and enhance tissue penetration [11–13]. Mechanistically, drug delivery from such systems is governed by crosslinked drug transport processes and steps that are further influenced by the local physiological environment. In particular, post-operative oedema [14, 15], commonly induced by surgical trauma, alters interstitial pressure, fluid flow, and transport resistance, thereby affecting drug distribution and availability. A quantitative understanding of how tissue conditions and nanocarrier properties regulate delivery outcomes is therefore critical for optimising therapeutic performance; however, this remains insufficiently explored.

Mathematical modelling provides a powerful framework to investigate coupled transport and reaction processes in drug delivery [16, 17]. By integrating governing equations describing mass transport, fluid flow, and biochemical interactions, such models enable systematic and reproducible evaluation of key parameters that are difficult to isolate experimentally. Foundational work established a transport framework for macromolecular delivery in solid tumours [17, 18], which was later adapted to study polymer-based drug delivery in idealised brain geometries [19–21]. More recently, 3-D models incorporating realistic brain anatomy have been developed to simulate wafer-based [22, 23] and convection-enhanced delivery [24, 25] in treatments against brain cancers. Our previous modelling work compared the performances of small-molecule drugs in hydrogel-mediated delivery to the post-operative brain [26] and assessed the impact of their transport properties [27]. However, a mechanistic assessment of how tissue properties and nanocarrier characteristics govern the outcomes of hydrogel-nanocarrier combined delivery systems remains lacking.

In this study, we employ a mechanistic mathematical model to simulate the delivery of nanocarrier-encapsulated anticancer agents from an implanted hydrogel into surrounding oedematous brain tissue under varying conditions. Simulations are conducted in a 3-D, patient-specific post-operative brain geometry, which is reconstructed from medical imaging data. The mechanistic model captures the key processes governing delivery, including nanocarrier and free drug transport from the hydrogel, interstitial transport within brain tissue, drug release, cellular uptake, and elimination via biochemical reactions and physical degradation, as well as the influence of oedema on the intratumoural environment. Delivery performance is quantitatively evaluated in terms of drug availability and spatial distribution, providing insights into the design of improved hydrogel-nanocarrier delivery systems.

## Materials and Methods

### Mathematical Model

The mathematical model consists of three linked mechanistic modules for interstitial fluid flow, interstitial transport of nanocarrier-encapsulated drugs and the drug after release from nanocarriers.

#### Transport Model of Interstitial Fluid

Since the distance between capillaries is commonly two orders of magnitude lower than the dimension of the brain, the brain tissue can be considered a porous medium in which the capillaries are distributed [17, 18]. Therefore, the flow of the incompressible interstitial fluid is described by the combination of the continuous equation and Darcy’s law, as

1 $$\nabla \cdot \mathbf{u}=L\frac{S}{V}\left[{p}_{\mathrm{b}}-p-\sigma {\pi}_{\mathrm{e}\mathrm{f}\mathrm{f}}\right]$$

2 $$\mathbf{u}=-\frac{\mu }{\kappa }\nabla p$$

where $\mathbf{u}$ and p are the flow velocity and pressure, respectively. ${p}_{\mathrm{b}}$ is the blood pressure. L refers to the hydraulic conductivity of the blood capillary wall. S/V is the surface area of capillaries in unit tissue volume. σ stands for the reflection coefficient of proteins in the blood plasma. ${\pi}_{\mathrm{e}\mathrm{f}\mathrm{f}}$ is the effective osmotic pressure, defined as the osmotic pressure difference across the blood vessel wall. μ is the dynamic viscosity of the interstitial fluid, and κ is the brain tissue’s permeability. Particularly, the right-hand-side term of Eq. (1) defines the plasma leakage rate from the blood lumen to the interstitial space, denoted by ${F}_{\mathrm{b},\mathrm{i}}$.

#### Transport model of therapeutic agents

**Fig. 1:**
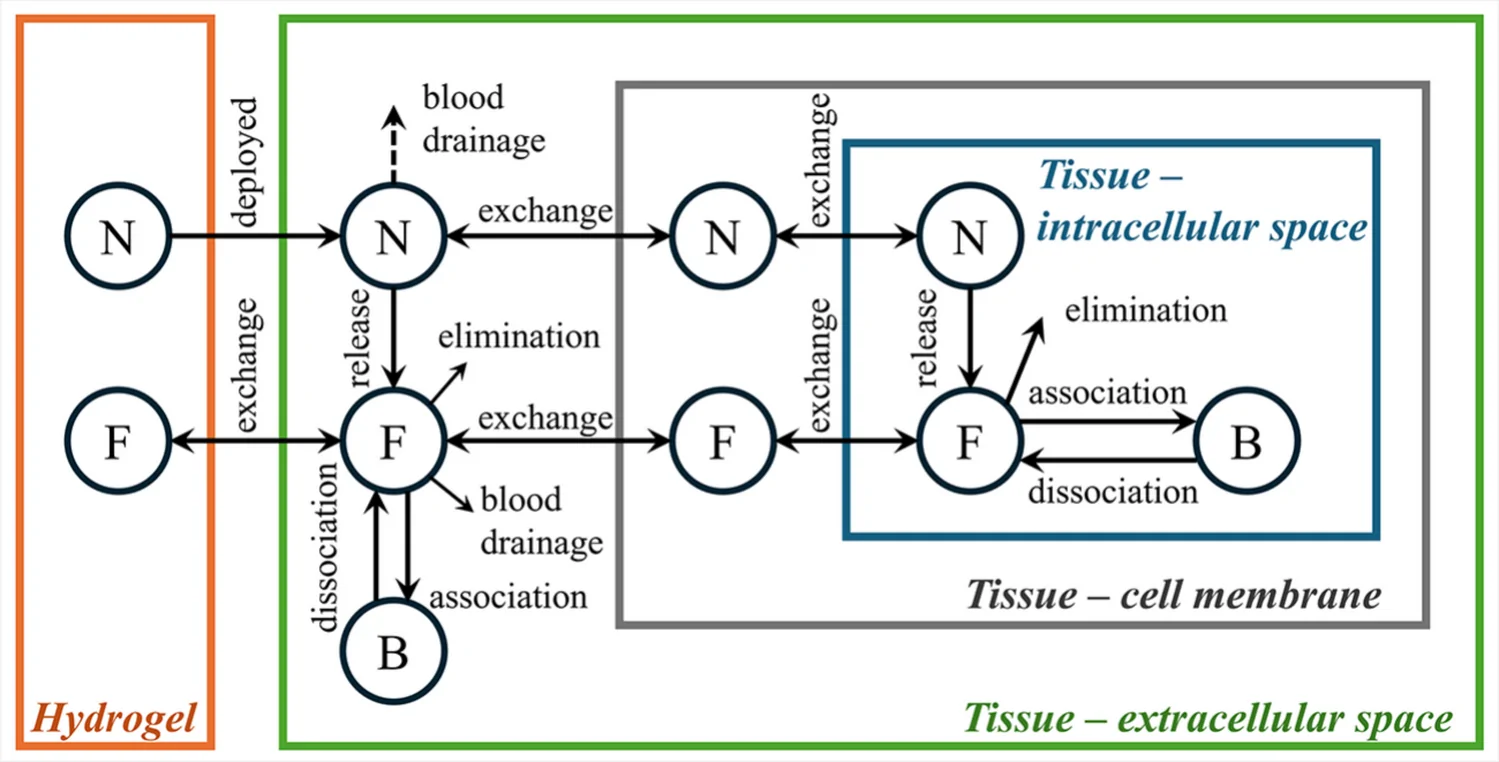
Drug transport processes in hydrogel-mediated nanocarrier delivery to the brain tissue. The letters N, F, and B denote nanocarriers, drugs in the free form, and protein-bound drugs, respectively. The nanocarrier loss due to blood drainage depends on their transvascular permeability, marked by the dashed line

Figure 1 schematically illustrates the drug transport steps involved in hydrogel-mediated nanocarrier delivery to brain tissue. Drug-loaded nanocarriers are incorporated into the hydrogel and deployed into the interstitial space of the surrounding brain tissue, where the loaded drugs can be released. The released free drugs can transfer back into the hydrogel driven by concentration gradients across the hydrogel surface, bind to proteins, undergo physical degradation or elimination due to bioreactions. Importantly, both nanocarriers and free drugs present in the tissue interstitial space can cross the cell membrane and enter the intracellular space. Within cells, drugs may also be released from nanocarriers, bind to intracellular proteins, and undergo elimination processes.

##### Nanocarrier-encapsulated Drug

Obeying the principle of conservation of mass, the nanocarrier concentration (${C}_{\mathrm{N},\mathrm{T}\mathrm{I}\mathrm{S}}$) in the brain tissue can be accounted for by

3 $${C}_{\mathrm{N},\mathrm{T}\mathrm{I}\mathrm{S}}={\nu}_{\mathrm{E}\mathrm{C}\mathrm{S}}{C}_{\mathrm{N},\mathrm{E}\mathrm{C}\mathrm{S}}+{\nu}_{\mathrm{C}\mathrm{M}}{C}_{\mathrm{N},\mathrm{C}\mathrm{M}}+{\nu}_{\mathrm{I}\mathrm{C}\mathrm{S}}{C}_{\mathrm{N},\mathrm{I}\mathrm{C}\mathrm{S}}$$

in which ν is the volume fraction, and C is the concentration. The subscripts ECS, CM, and ICS denote the extracellular space, cell membrane, and intracellular space, respectively, which together constitute the three compartments of brain tissue. The subscript N refers to nanocarriers.

The nanocarriers travel in the brain tissues by convection and diffusion, internalisation, drug release in the extracellular and intracellular space, and possible loss to the blood, as

4 $$\begin{aligned}\frac{\partial {C}_{\mathrm{N},\mathrm{T}\mathrm{I}\mathrm{S}}}{\partial t}&={D}_{\mathrm{N},\mathrm{E}\mathrm{C}\mathrm{S}}{\nabla }^{2}\left({\nu}_{\mathrm{E}\mathrm{C}\mathrm{S}}{C}_{\mathrm{N},\mathrm{E}\mathrm{C}\mathrm{S}}\right)-\nabla \cdot \left(\mathbf{u}{\nu}_{\mathrm{E}\mathrm{C}\mathrm{S}}{C}_{\mathrm{N},\mathrm{E}\mathrm{C}\mathrm{S}}\right)\\ &-{k}_{\mathrm{r}\mathrm{e}\mathrm{l}}{\nu}_{\mathrm{E}\mathrm{C}\mathrm{S}}{C}_{\mathrm{N},\mathrm{E}\mathrm{C}\mathrm{S}}-{k}_{\mathrm{r}\mathrm{e}\mathrm{l}}{\nu}_{\mathrm{I}\mathrm{C}\mathrm{S}}{C}_{\mathrm{N},\mathrm{I}\mathrm{C}\mathrm{S}}-{k}_{\mathrm{b},\mathrm{N}}{\nu}_{\mathrm{E}\mathrm{C}\mathrm{S}}{C}_{\mathrm{N},\mathrm{E}\mathrm{C}\mathrm{S}}\end{aligned}$$

where ${D}_{\mathrm{N},\mathrm{E}\mathrm{C}\mathrm{S}}$ is the diffusivity of nanocarriers in the tissue extracellular space. ${k}_{\mathrm{r}\mathrm{e}\mathrm{l}}$ is the drug release rate. ${k}_{\mathrm{b}}$ is the rate of loss to the blood.

Given the time window of hydrogel-mediated delivery commonly spans weeks and even months, which is higher than the nanocarrier internalisation rate [28], it is assumed that dynamic equilibrium is reached between the nanocarrier concentration in the intracellular and extracellular space, and between the cell membrane and extracellular space, as ${P}_{\mathrm{N},\mathrm{I}\mathrm{C}\mathrm{S}-\mathrm{E}\mathrm{C}\mathrm{S}}={C}_{\mathrm{N},\mathrm{I}\mathrm{C}\mathrm{S}}/{C}_{\mathrm{N},\mathrm{E}\mathrm{C}\mathrm{S}}$ and ${P}_{\mathrm{N},\mathrm{C}\mathrm{M}-\mathrm{E}\mathrm{C}\mathrm{S}}={C}_{\mathrm{N},\mathrm{C}\mathrm{M}}/{C}_{\mathrm{N},\mathrm{E}\mathrm{C}\mathrm{S}}$, where ${P}_{\mathrm{N},\mathrm{I}\mathrm{C}\mathrm{S}-\mathrm{E}\mathrm{C}\mathrm{S}}$ and ${P}_{\mathrm{N},\mathrm{C}\mathrm{M}-\mathrm{E}\mathrm{C}\mathrm{S}}$ are the apparent partitioning coefficient of nanocarriers between the intracellular and extracellular space, and the cell membrane and extracellular space, respectively. No nanocarriers are permanently bound to or release the payload on the cell membrane. Therefore, Eq. (4) can be simplified as

5 $$\begin{aligned}\frac{\partial {C}_{\mathrm{N},\mathrm{E}\mathrm{C}\mathrm{S}}}{\partial t}&={D}_{\mathrm{N},\mathrm{E}\mathrm{C}\mathrm{S}}^{*}{\nabla }^{2}{C}_{\mathrm{N},\mathrm{E}\mathrm{C}\mathrm{S}}-{\mathbf{u}}_{\mathrm{N}}^{*}\cdot \nabla {C}_{\mathrm{N},\mathrm{E}\mathrm{C}\mathrm{S}}\\ &-{k}_{\mathrm{r}\mathrm{e}\mathrm{l},\mathrm{N}}^{*}{C}_{\mathrm{N},\mathrm{E}\mathrm{C}\mathrm{S}}-{k}_{\mathrm{e}\mathrm{l}\mathrm{m},\mathrm{N}}^{*}{C}_{\mathrm{N},\mathrm{E}\mathrm{C}\mathrm{S}}\end{aligned}$$

in which ${D}_{\mathrm{N},\mathrm{E}\mathrm{C}\mathrm{S}}^{*}={D}_{\mathrm{N},\mathrm{E}\mathrm{C}\mathrm{S}}{\nu}_{\mathrm{E}\mathrm{C}\mathrm{S}}/{G}_{\mathrm{N}}$ is the apparent diffusion coefficient of nanocarriers in the tissue extracellular space. ${\mathbf{u}}_{\mathrm{N}}^{*}=\mathbf{u}{\nu}_{\mathrm{E}\mathrm{C}\mathrm{S}}/{G}_{\mathrm{N}}$ is the apparent velocity of interstitial fluid flow in terms of nanocarrier transport. ${k}_{\mathrm{r}\mathrm{e}\mathrm{l},\mathrm{N}}^{*}={k}_{\mathrm{r}\mathrm{e}\mathrm{l}}\left({\nu}_{\mathrm{E}\mathrm{C}\mathrm{S}}+{\nu}_{\mathrm{I}\mathrm{C}\mathrm{S}}{P}_{\mathrm{N},\mathrm{I}\mathrm{C}\mathrm{S}-\mathrm{E}\mathrm{C}\mathrm{S}}\right)/{G}_{\mathrm{N}}$ is the apparent release rate in terms of nanocarrier transport. ${k}_{\mathrm{e}\mathrm{l}\mathrm{m},\mathrm{N}}^{*}=\left({k}_{\mathrm{b},\mathrm{N}}+{F}_{\mathrm{b},\mathrm{i}}\right){\nu}_{\mathrm{E}\mathrm{C}\mathrm{S}}/{G}_{\mathrm{N}}$ is the nanocarrier’s apparent elimination rate. The parameter ${G}_{\mathrm{N}}={\nu}_{\mathrm{E}\mathrm{C}\mathrm{S}}+{\nu}_{\mathrm{C}\mathrm{M}}{P}_{\mathrm{N},\mathrm{C}\mathrm{M}-\mathrm{E}\mathrm{C}\mathrm{S}}+{\nu}_{\mathrm{I}\mathrm{C}\mathrm{S}}{P}_{\mathrm{N},\mathrm{I}\mathrm{C}\mathrm{S}-\mathrm{E}\mathrm{C}\mathrm{S}}$ is determined by the properties of the brain tissue and the nanocarrier, where ${\nu}_{\mathrm{C}\mathrm{M}}=1-{\nu}_{\mathrm{E}\mathrm{C}\mathrm{S}}-{\nu}_{\mathrm{I}\mathrm{C}\mathrm{S}}$.

##### Released Drug

In accordance with the principle of conservation of mass, the concentration of free drugs (${C}_{\mathrm{F},\mathrm{T}\mathrm{I}\mathrm{S}}$) and protein-bound drugs (${C}_{\mathrm{B},\mathrm{T}\mathrm{I}\mathrm{S}}$) in the brain tissue can be calculated by

6 $$\begin{array}{c}{C}_{\mathrm{F},\mathrm{T}\mathrm{I}\mathrm{S}}={\nu}_{\mathrm{E}\mathrm{C}\mathrm{S}}{C}_{\mathrm{F},\mathrm{E}\mathrm{C}\mathrm{S}}+{\nu}_{\mathrm{C}\mathrm{M}}{C}_{\mathrm{F},\mathrm{C}\mathrm{M}}+{\nu}_{\mathrm{I}\mathrm{C}\mathrm{S}}{C}_{\mathrm{F},\mathrm{I}\mathrm{C}\mathrm{S}}\\ {C}_{\mathrm{B},\mathrm{T}\mathrm{I}\mathrm{S}}={\nu}_{\mathrm{E}\mathrm{C}\mathrm{S}}{C}_{\mathrm{B},\mathrm{E}\mathrm{C}\mathrm{S}}+{\nu}_{\mathrm{C}\mathrm{M}}{C}_{\mathrm{B},\mathrm{C}\mathrm{M}}+{\nu}_{\mathrm{I}\mathrm{C}\mathrm{S}}{C}_{\mathrm{B},\mathrm{I}\mathrm{C}\mathrm{S}}\end{array}$$

The availability of the released drug in the brain tissue depends on its convective and diffusive transport, drug release from nanocarriers, elimination due to blood drainage, physical degradation, bioreactions, binding to proteins, and internalisation. Therefore, the drug concentration can be calculated by

7 $$\begin{aligned}\frac{\partial {C}_{\mathrm{F},\mathrm{T}\mathrm{I}\mathrm{S}}}{\partial t}&={D}_{\mathrm{F},\mathrm{E}\mathrm{C}\mathrm{S}}{\nabla }^{2}\left({\nu}_{\mathrm{E}\mathrm{C}\mathrm{S}}{C}_{\mathrm{F},\mathrm{E}\mathrm{C}\mathrm{S}}\right)-\nabla\cdot \left({\boldsymbol{u}}{\nu}_{\mathrm{E}\mathrm{C}\mathrm{S}}{C}_{\mathrm{F},\mathrm{E}\mathrm{C}\mathrm{S}}\right)\\ &+{k}_{\mathrm{r}\mathrm{e}\mathrm{l}}\left({\nu}_{\mathrm{E}\mathrm{C}\mathrm{S}}{C}_{\mathrm{N},\mathrm{E}\mathrm{C}\mathrm{S}}+{\nu}_{\mathrm{I}\mathrm{C}\mathrm{S}}{C}_{\mathrm{N},\mathrm{I}\mathrm{C}\mathrm{S}}\right)\\ &-{\nu}_{\mathrm{E}\mathrm{C}\mathrm{S}}\left({k}_{\mathrm{b},\mathrm{F}}+{k}_{\mathrm{e},\mathrm{F}}\right){C}_{\mathrm{F},\mathrm{E}\mathrm{C}\mathrm{S}}-{\nu}_{\mathrm{I}\mathrm{C}\mathrm{S}}{k}_{\mathrm{e},\mathrm{F}}{C}_{\mathrm{F},\mathrm{I}\mathrm{C}\mathrm{S}}-\frac{\partial {C}_{\mathrm{B},\mathrm{T}\mathrm{I}\mathrm{S}}}{\partial t}\end{aligned}$$

in which ${D}_{\mathrm{F},\mathrm{E}\mathrm{C}\mathrm{S}}$ is the diffusivity of the free drug in the extracellular space. ${k}_{\mathrm{e},\mathrm{F}}$ is the drug elimination rate, combining the contribution of degradation and bioreaction. The subscript F and B denote free drug and protein-bound drug, respectively.

Two assumptions are further introduced. (1) The drug binding to proteins takes place rapidly as compared to the delivery time window. Therefore, the concentration of protein-bound drug is proportional to that of free drug in each compartment [22], as ${K}_{\mathrm{E}\mathrm{C}\mathrm{S}}={C}_{\mathrm{B},\mathrm{E}\mathrm{C}\mathrm{S}}/{C}_{\mathrm{F},\mathrm{E}\mathrm{C}\mathrm{S}}$ and ${K}_{\mathrm{I}\mathrm{C}\mathrm{S}}={C}_{\mathrm{B},\mathrm{I}\mathrm{C}\mathrm{S}}/{C}_{\mathrm{F},\mathrm{I}\mathrm{C}\mathrm{S}}$. (2) Dynamic equilibrium has been reached for drug concentration in the intra- and extra-cellular concentrations [21], as ${P}_{\mathrm{F},\mathrm{I}\mathrm{C}\mathrm{S}-\mathrm{E}\mathrm{C}\mathrm{S}}={C}_{\mathrm{F},\mathrm{I}\mathrm{C}\mathrm{S}}/{C}_{\mathrm{F},\mathrm{E}\mathrm{C}\mathrm{S}}$ and ${P}_{\mathrm{F},\mathrm{C}\mathrm{M}-\mathrm{E}\mathrm{C}\mathrm{S}}={C}_{\mathrm{F},\mathrm{C}\mathrm{M}}/{C}_{\mathrm{F},\mathrm{E}\mathrm{C}\mathrm{S}}$; ${P}_{\mathrm{F},\mathrm{I}\mathrm{C}\mathrm{S}-\mathrm{E}\mathrm{C}\mathrm{S}}$ and ${P}_{\mathrm{F},\mathrm{C}\mathrm{M}-\mathrm{E}\mathrm{C}\mathrm{S}}$ are the partitioning coefficient of free drug between the intra- and extra-cellular space, and the cell membrane and extracellular space, respectively. Moreover, no drug is either bound or being eliminated on the cell membrane [21]. Therefore, Eq. (7) can be rewritten as

8 $$\begin{aligned}\frac{\partial {C}_{\mathrm{F},\mathrm{E}\mathrm{C}\mathrm{S}}}{\partial t}&={D}_{\mathrm{F},\mathrm{E}\mathrm{C}\mathrm{S}}^{*}{\nabla }^{2}{C}_{\mathrm{F},\mathrm{E}\mathrm{C}\mathrm{S}}-{\mathbf{u}}_{\mathrm{F}}^{*}\cdot \nabla {C}_{\mathrm{F},\mathrm{E}\mathrm{C}\mathrm{S}}\\ &+{k}_{\mathrm{r}\mathrm{e}\mathrm{l},\mathrm{F}}^{*}{C}_{\mathrm{N},\mathrm{E}\mathrm{C}\mathrm{S}}-{k}_{\mathrm{e}\mathrm{l}\mathrm{m},\mathrm{F}}^{*}{C}_{\mathrm{F},\mathrm{E}\mathrm{C}\mathrm{S}}\end{aligned}$$

where ${D}_{\mathrm{F},\mathrm{E}\mathrm{C}\mathrm{S}}^{*}={\nu}_{\mathrm{E}\mathrm{C}\mathrm{S}}{D}_{\mathrm{F},\mathrm{E}\mathrm{C}\mathrm{S}}/{G}_{\mathrm{F}}$ is the apparent diffusion coefficient of free drugs in the tissue extracellular space. ${\mathbf{u}}_{\mathrm{F}}^{*}=\mathbf{u}{\nu}_{\mathrm{E}\mathrm{C}\mathrm{S}}/{G}_{\mathrm{F}}$ is the apparent velocity of interstitial fluid flow in terms of free drug transport. ${k}_{\mathrm{r}\mathrm{e}\mathrm{l},\mathrm{F}}^{*}={k}_{\mathrm{r}\mathrm{e}\mathrm{l}}\left({\nu}_{\mathrm{E}\mathrm{C}\mathrm{S}}+{\nu}_{\mathrm{I}\mathrm{C}\mathrm{S}}{P}_{\mathrm{N},\mathrm{I}\mathrm{C}\mathrm{S}-\mathrm{E}\mathrm{C}\mathrm{S}}\right)/{G}_{\mathrm{F}}$ is the apparent release rate in terms of free drug transport. ${k}_{\mathrm{e}\mathrm{l}\mathrm{m},\mathrm{F}}^{*}={k}_{\mathrm{e}\mathrm{l}\mathrm{m},\mathrm{F}}/{G}_{\mathrm{F}}=\left[{\nu}_{\mathrm{E}\mathrm{C}\mathrm{S}}{k}_{\mathrm{b},\mathrm{F}}+\left({\nu}_{\mathrm{E}\mathrm{C}\mathrm{S}}+{\nu}_{\mathrm{I}\mathrm{C}\mathrm{S}}\right){k}_{\mathrm{e},\mathrm{F}}+{\nu}_{\mathrm{E}\mathrm{C}\mathrm{S}}{F}_{\mathrm{b},\mathrm{i}}\right]/{G}_{\mathrm{F}}$ is the apparent elimination rate of the free drug. The parameter ${G}_{\mathrm{F}}={\nu}_{\mathrm{E}\mathrm{C}\mathrm{S}}\left(1+{K}_{\mathrm{E}\mathrm{C}\mathrm{S}}\right)+{\nu}_{\mathrm{I}\mathrm{C}\mathrm{S}}{P}_{\mathrm{F},\mathrm{I}\mathrm{C}\mathrm{S}-\mathrm{E}\mathrm{C}\mathrm{S}}\left(1+{K}_{\mathrm{I}\mathrm{C}\mathrm{S}}\right)+\left(1-{\nu}_{\mathrm{E}\mathrm{C}\mathrm{S}}-{\nu}_{\mathrm{I}\mathrm{C}\mathrm{S}}\right){P}_{\mathrm{F},\mathrm{C}\mathrm{M}-\mathrm{E}\mathrm{C}\mathrm{S}}$ is subject to the properties of the brain tissue and the free drug.

The free drug after release in the tissue can transfer back into the hydrogel, where the drug moves by diffusion. The hydrogel is also assumed to be inert to the reaction with the drug. Therefore, the concentration of free drug in the hydrogel (${C}_{\mathrm{F},\mathrm{G}\mathrm{E}\mathrm{L}}$) can be obtained by

9 $$\frac{\partial {C}_{\mathrm{F},\mathrm{G}\mathrm{E}\mathrm{L}}}{\partial t}={D}_{\mathrm{F},\mathrm{G}\mathrm{E}\mathrm{L}}{\nabla }^{2}{C}_{\mathrm{F},\mathrm{G}\mathrm{E}\mathrm{L}}$$

in which ${D}_{\mathrm{F},\mathrm{G}\mathrm{E}\mathrm{L}}$ is the diffusion coefficient of the free drug in the hydrogel.

### Model Geometry

**Fig. 2:**
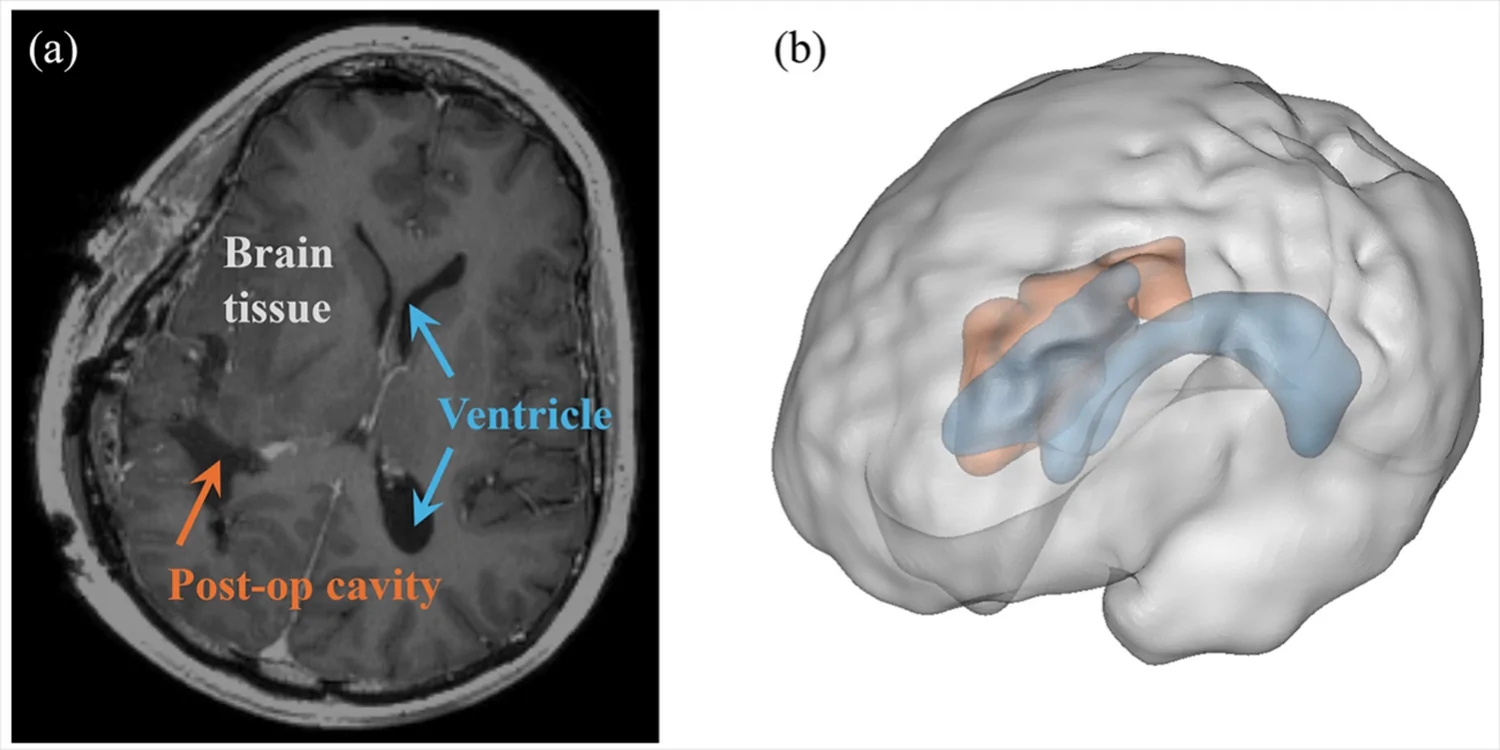
Model geometry. (**a**) An example MRI slice shows the brain parenchyma, ventricle, and post-surgery cavity. (**b**) the 3-D computational domain. The pale blue region is the brain ventricle. The orange region is the cavity left after the tumour resection and filled with the hydrogel, which is loaded with a nanocarrier-encapsulated drug.

The 3-D post-operative brain geometry, including the ventricle and the cavity remaining after tumour resection, is reconstructed from the anonymised patient's Magnetic Resonance Imaging (MRI) data stored in the TCIA database [29]. Each MRI slice has a thickness of 1 mm and a resolution of 256 × 256 pixels, with a pixel size of 0.938 mm. An example slice is shown in Fig. 2a.

The ventricle and post-operative cavity are segmented from the brain parenchyma on each image slice based on local signal intensity using 3D Slicer. The resulting surfaces are smoothed and imported into COMSOL Multiphysics to generate the computational mesh. Following a mesh convergence study, the final mesh contains approximately 8.7 million tetrahedral elements. To accurately resolve transport near the hydrogel-tissue interface, a minimum element size of 0.1 mm is employed in this region. The reconstructed geometry model is given in Fig. 2b. The total volumes of the brain and the cavity are 1434 mL and 24 mL, respectively.

### Model Parameters

Since the time window of drug transport is usually much shorter than the tissue growth, the tissue and drug properties are considered constant over time. The baseline parameter values are listed in Tables I and II for the brain tissue and therapeutic agents, respectively. The rationales for selecting the key parameters are explained as follows.

**Table I:** Tissue-related Model Parameters

Symbol	Parameter	Unit	Value
${\nu}_{\mathrm{E}\mathrm{C}\mathrm{S}}$	Tissue porosity	-	0.20 [19, 23]
${\nu}_{\mathrm{I}\mathrm{C}\mathrm{S}}$	Volume fraction of intracellular space	-	0.65 [19, 23]
ρ	Density of the interstitial fluid	$\mathrm{k}\mathrm{g}/{\mathrm{m}}^{3}$	1000 [19, 23]
μ	Dynamic viscosity of the interstitial fluid	$\mathrm{P}\mathrm{a}\bullet \mathrm{s}$	0.00078 [30, 31]
${\pi}_{\mathrm{e}\mathrm{f}\mathrm{f}}$	Transvascular osmotic pressure difference	$\mathrm{P}\mathrm{a}$	2700 [23, 32]
${p}_{\mathrm{b}}$	Blood pressure	$\mathrm{P}\mathrm{a}$	4610 [23, 32]
S/V	Surface area of blood vessel wall per tissue volume	${\mathrm{m}}^{-1}$	7000 [23, 32]
σ	Reflection coefficient for blood proteins	-	0.91 [32]
${L}_{0}$	Initial hydraulic conductivity of blood vessel walls	$\mathrm{m}/\mathrm{P}\mathrm{a}/\mathrm{s}$	$1.4\times {10}^{-13}$ [23]
κ	Tissue permeability	${\mathrm{m}}^{2}$	$1.0\times {10}^{-15}$*

*The parameter value of tissue permeability is justified in this study

**Table II:** Drug-related Model Parameters*

Symbol	Parameter	Unit	Nanocarrier	Paclitaxel
${P}_{\mathrm{C}\mathrm{M}-\mathrm{E}\mathrm{C}\mathrm{S}}$	Cell membrane and extracellular space partition coefficient	-	1.0	3162.3 [20, 23]
${P}_{\mathrm{I}\mathrm{C}\mathrm{S}-\mathrm{E}\mathrm{C}\mathrm{S}}$	Intra- and extra-cellular space partition coefficient	-	1.0	1.0 [19]
${K}_{\mathrm{E}\mathrm{C}\mathrm{S}}$*,* ${K}_{\mathrm{I}\mathrm{C}\mathrm{S}}$	Ratio of protein-bound drug to free drug	-	-	5.1 [23, 33]
${D}_{\mathrm{G}\mathrm{E}\mathrm{L}}$	Diffusion coefficient in the hydrogel	${\mathrm{m}}^{2}/\mathrm{s}$	-	$1.6\times {10}^{-10}$ [34]
${D}_{\mathrm{E}\mathrm{C}\mathrm{S}}$	Diffusion coefficient in the tissue extracellular space	${\mathrm{m}}^{2}/\mathrm{s}$	$1.0\times {10}^{-12}$	$1.1\times {10}^{-10}$ [35]
${k}_{\mathrm{r}\mathrm{e}\mathrm{l}}$	Drug release rate from the nanocarriers	${\mathrm{s}}^{-1}$	$1.0\times {10}^{-4}$	-
${k}_{\mathrm{b}}$	Loss rate due to blood drainage	${\mathrm{s}}^{-1}$	$1.0\times {10}^{-7}$	$1.4\times {10}^{-4}$ [23]
${k}_{\mathrm{e}}$	Combined drug elimination rate	${\mathrm{s}}^{-1}$	-	$6.8\times {10}^{-7}$ [23]

*The parameter values of nanocarrier properties are justified in this study

#### Hydraulic Conductivity of Capillary Wall

Surgical intervention can cause trauma to the brain tissue surrounding the glioblastoma, leading to damage to the capillary walls. Such damage presents as an increase in permeability of capillary vessel walls to plasma, which is widely recognised as a primary mechanism underlying vasogenic oedema. This alteration in vascular function can be quantitatively characterised by the hydraulic conductivity of the capillary wall. MRI-based analysis suggested that the hydraulic conductivity of the capillary wall can increase by two orders of magnitude under oedematous conditions [36, 37]. The volume transfer constant, ${K}^{\mathrm{T}\mathrm{r}\mathrm{a}\mathrm{n}\mathrm{s}}$, has been reported to be on the order of $O\left(-4\right) {\mathrm{m}\mathrm{i}\mathrm{n}}^{-1}$ to $O\left(-3\right) {\mathrm{m}\mathrm{i}\mathrm{n}}^{-1}$ in normal brain tissue with an intact blood–brain barrier (BBB) [38], whereas values on the order of $O\left(-2\right) {\mathrm{m}\mathrm{i}\mathrm{n}}^{-1}$ to $O\left(-1\right) {\mathrm{m}\mathrm{i}\mathrm{n}}^{-1}$ have been measured in brain tissues with a disrupted BBB [39]. These observations suggest that the hydraulic conductivity may increase by up to three orders of magnitude under severe pathological conditions. Moreover, ${K}^{\mathrm{T}\mathrm{r}\mathrm{a}\mathrm{n}\mathrm{s}}$ was reported to be close to zero in pre-operative brain, while the value increased to $O\left(-2\right) {\mathrm{m}\mathrm{i}\mathrm{n}}^{-1}$ after the operation [40]. Therefore, an upper bound corresponding to a 1000-fold increase in hydraulic conductivity is considered. To comprehensively investigate its impact, a broad range of enhancement factors from 1 to 1000 is examined, with a baseline value of 100 [41].

#### Tissue Permeability

Tissue permeability characterises the ease with which interstitial fluid perfuses through the brain parenchyma. Values reported for brain tissue vary widely in the literature. For instance, a permeability of $7.1\times {10}^{-15}\hspace{0.17em}{\mathrm{m}}^{2}$ was applied to the brain tissue [23], whereas a much smaller value of $2.47\times {10}^{-17}\hspace{0.17em}{\mathrm{m}}^{2}$ could be found in other studies [42, 43]. Model-based analyses suggested that the permeability is on the order of $O({10}^{-16})\hspace{0.17em}{\mathrm{m}}^{2}$ [44], while experimental measurements indicated a range from $O({10}^{-17})\hspace{0.17em}{\mathrm{m}}^{2}$ to $O({10}^{-15})\hspace{0.17em}{\mathrm{m}}^{2}$ [45]. Based on these findings, a range of $1.0\times {10}^{-17} {\mathrm{m}}^{2}$ to $1.0\times {10}^{-14}\hspace{0.17em}{\mathrm{m}}^{2}$ is considered in the present study, with a baseline value of $1.0\times {10}^{-15}\hspace{0.17em}{\mathrm{m}}^{2}$.

#### Diffusion Coefficients

Diffusion coefficient represents the ability of therapeutic agents to travel in the transport medium. It is subject to the properties of the therapeutic agents, including the size and surface charge, and the brain tissue properties, including the porosity, tortuosity, and fluid viscosity. The diffusion coefficient of paclitaxel in the brain tissue is calculated based on its molecular weight (MW) of $853.91 \mathrm{D}\mathrm{a}$ [46], using the empirical relationship [35] of

10 $${D}_{\mathrm{F},\mathrm{E}\mathrm{C}\mathrm{S}}=1.778\times {10}^{-8}{\left(MW\right)}^{-0.75}$$

The value is calculated as $1.1\times {10}^{-10} {\mathrm{m}}^{2}/\mathrm{s}$. The diffusivity of paclitaxel in hydrogels was reported to be $1.6\times {10}^{-10} {\mathrm{m}}^{2}/\mathrm{s}$ [34].

The diffusion coefficient of different nanocarriers in the brain was measured $5.36\times {10}^{-11} {\mathrm{m}}^{2}/\mathrm{s}$, $6.48\times {10}^{-12} {\mathrm{m}}^{2}/\mathrm{s}$, and $1.67\times {10}^{-13} {\mathrm{m}}^{2}/\mathrm{s}$ for nanocarriers with a dimension of 3 nm, 14.1 nm, and 35.4 nm [47]. The diffusivity of $2.43\times {10}^{-13} {\mathrm{m}}^{2}/\mathrm{s}$ [48] and $1.0\times {10}^{-14} {\mathrm{m}}^{2}/\mathrm{s}$ [49] were applied for a 100-nm and 500-nm nanocarrier, respectively. For a nanocarrier whose size could vary during the delivery process, its diffusivity was found to be $2.3\times {10}^{-11} {\mathrm{m}}^{2}/\mathrm{s}$ and $2.2\times {10}^{-12} {\mathrm{m}}^{2}/\mathrm{s}$ [50]. Moreover, a diffusivity of $7.7\times {10}^{-14} {\mathrm{m}}^{2}/\mathrm{s}$ was reported for a 50 nm nanocarrier [51]. To cover these values, a range of $1.0\times {10}^{-14} {\mathrm{m}}^{2}/\mathrm{s}$ to $1.0\times {10}^{-10} {\mathrm{m}}^{2}/\mathrm{s}$ is employed, with the baseline value set to be $1.0\times {10}^{-12} {\mathrm{m}}^{2}/\mathrm{s}$.

#### Blood Drainage Rate

The speed of therapeutic agents to traverse the blood capillary wall to lost into the systemic circulation can be quantified by the blood drainage rate. Due to the nearly impermeable nature of the BBB, most substances are unable to cross the wall of continuous blood capillaries in the brain. However, evidence shows that paclitaxel presents a significant ability to penetrate the BBB [52]. Studies also demonstrate that nanocarriers can cross the wall of brain blood vessels with surface modifications using various ligands [53]. Their transvascular permeability was reported in the range of $O\left({10}^{-11}\right) \mathrm{m}/\mathrm{s}$ to $O\left({10}^{-7}\right) \mathrm{m}/\mathrm{s}$ [54, 55]. Given that the surface area of the capillary wall per tissue volume is around $7000 {\mathrm{m}}^{-1}$ [22, 23], the blood drainage rate, which is defined as the product of the vascular permeability and surface are of the capillary wall per tissue volume, ranges from 0 to $1.0\times {10}^{-3} {\mathrm{s}}^{-1}$. The minimal represents the nanocarriers which are unable to penetrate the BBB. The baseline value of $1.0\times {10}^{-7} {\mathrm{s}}^{-1}$ is set in this study.

#### Apparent Partition Coefficients of Nanocarriers

The partition coefficient between the cell membrane and tissue extracellular space is defined as the ratio of nanocarrier concentration in the lipid bilayer of the cell membrane to that in the brain tissue extracellular space, which represents an aqueous phase. Reported values for analogous systems, such as the partition coefficient between water and octanol, range from 0.07 to 27.78 in the literature [56, 57]. A baseline value of 1.0 is assumed. To evaluate its influence on model outcomes, a sensitivity analysis is performed by varying this parameter over a range of 0.01–100.

Nanocarrier uptake by cells may involve multiple internalisation mechanisms, including macropinocytosis, clathrin-mediated endocytosis, caveolae-dependent endocytosis, and adsorptive-mediated endocytosis. These processes can involve interactions between ligands on functionalised nanocarrier surface and specific membrane receptors, as well as active transport steps that require ATP. On the other hand, nanocarrier exocytosis proceeds via multiple pathways, including passive diffusion, rapid recycling, lysosomal secretion, and ER-Golgi-mediated vesicular transport. The apparent partition coefficient describes the ratio of concentration in the intracellular and extracellular space when dynamic equilibrium has been reached. Given the limited availability of quantitative data for this parameter, the baseline of the apparent partition coefficient between the intracellular and extracellular spaces is set to 1.0, as both compartments are aqueous phases. The same range of 0.01–100 is adopted to examine its impact on delivery outcomes.

#### Drug Release Rate

The release rate of drugs from nanocarriers characterises the intrinsic timescale over which nanocarriers release their encapsulated payload, with larger values corresponding to faster drug release. As a key parameter governing therapeutic efficacy [58, 59], it can change over a wide range depending on formulation, fabrication strategy, and environmental conditions [60, 61]. For example, light-responsive nanoparticles may complete drug release within minutes to hours [62, 63], whereas thermosensitive liposomes can discharge their cargo within seconds once the temperature exceeds a phase-transition threshold [64]. In comparison, stealth liposomes can provide sustained release over several days to weeks [65]. Using the first-order release kinetic model [66], the corresponding release rate is located in the range from $1.0\times {10}^{-6}$ ${\mathrm{s}}^{-1}$ to $1.0\times {10}^{-2} {\mathrm{s}}^{-1}$ [25]. To encompass the plausible magnitudes reported in the literature, the same range is adopted to evaluate its impact. The baseline value is $1.0\times {10}^{-4} {\mathrm{s}}^{-1}$, representing an intermediate release timescale.

#### Drug Dose

The reported loading dose of paclitaxel varies considerably across studies. For example, a loading concentration of 3 mg/mL [67] has been reported in one study, whereas another study used 30 mg/mL [68]. In addition, a separate study reported that 20 mg of hydrogel contained 5.4 mg of paclitaxel [69], corresponding to a dose of 270 mg/mL. To encompass the range reported in the literature, a baseline loading concentration of 30 mg/mL is selected. The drug dose is then varied over a range from 3 mg/mL to 300 mg/mL to investigate its impact on drug delivery outcomes.

#### Blood Pressure

The normal range of blood pressure is measured in the range from 3200 to 4700 Pa [32]. This range is applied in the following parametric study to explore its impact on the delivery outcomes.

### Numerical Methods

The governing equations are solved using the finite element method-based software COMSOL Multiphysics. Given that hydrogel-mediated drug delivery and oedema evolution may persist for days to weeks, representing a relatively slow process, quasi-steady-state simulations are performed.

### Boundary Conditions

A pressure of 657.9 Pa [70] is imposed on the brain surface, while the pressure on the ventricular surface is specified as 500 Pa [71]. Since both surfaces are connected to the cerebrospinal fluid circulation, they are treated as sinks, where the drug concentration is assumed to be zero. A prescribed nanocarrier concentration is applied at the hydrogel boundary to represent drug administration from the hydrogel into the brain tissue. The flux of free drug across the interface satisfies

11 $$\begin{array}{l}-{D}_{\mathrm{F},\mathrm{G}\mathrm{E}\mathrm{L}}\frac{\partial {C}_{\mathrm{F},\mathrm{G}\mathrm{E}\mathrm{L}}}{\partial n}=-{D}_{\mathrm{F},\mathrm{E}\mathrm{C}\mathrm{S}}\frac{\partial {C}_{\mathrm{F}, \mathrm{E}\mathrm{C}\mathrm{S}}}{\partial n},\\ {C}_{\mathrm{F},\mathrm{G}\mathrm{E}\mathrm{L}}={P}_{\mathrm{F},\mathrm{G}\mathrm{E}\mathrm{L}-\mathrm{E}\mathrm{C}\mathrm{S}}{C}_{\mathrm{F},\mathrm{E}\mathrm{C}\mathrm{S}}\end{array}$$

where ${P}_{\mathrm{F},\mathrm{G}\mathrm{E}\mathrm{L}-\mathrm{E}\mathrm{C}\mathrm{S}}$ denotes the partition coefficient of the free drug between the hydrogel and brain tissue. It is assumed to be unity [22, 23], since both the tissue and the hydrogel can be considered aqueous phases.

### Qualification Indicators

#### Drug Availability

Drug availability (${A}_{\mathrm{d}}$) describes the amount of drug that accumulates in the brain tissue. It can be defined as

12 $${A}_{\mathrm{d}}=\sum {C}_{\mathrm{E}\mathrm{C}\mathrm{S},\mathrm{i}}{V}_{\mathrm{i}}$$

in which ${C}_{\mathrm{E}\mathrm{C}\mathrm{S},\mathrm{i}}$ is the local concentration in the tissue extracellular space, and ${V}_{\mathrm{i}}$ is the corresponding tissue volume.

#### Distribution Volume

Distribution volume (${V}_{\mathrm{d}\mathrm{i}\mathrm{s}}$) is applied to evaluate the penetration of the free drug into the brain tissue, defined as

13 $$\begin{array}{cc}{V}_{\mathrm{d}\mathrm{i}\mathrm{s}}=\sum {V}_{\mathrm{i}}& \left({C}_{\mathrm{F},\mathrm{E}\mathrm{C}\mathrm{S},\mathrm{i}}\ge 0.01\%{C}_{\mathrm{N},\mathrm{G}\mathrm{E}\mathrm{L},0}\right)\end{array}$$

${C}_{\mathrm{N},\mathrm{G}\mathrm{E}\mathrm{L},0}$ stands for the baseline concentration of nanocarrier-encapsulated drug at the implemented hydrogel surface, set as 30 mg/mL [68]. The threshold of 0.01% [27] is selected to account for the distribution volume.

#### Distribution Heterogeneity

Distribution heterogeneity (${H}_{\mathrm{d}}$) evaluates the degree of spatial variation in drug concentration within the tissue. It can be defined as

14 $${H}_{\mathrm{d}}=\frac{\sum \left|{C}_{\mathrm{F},\mathrm{E}\mathrm{C}\mathrm{S},\mathrm{i}}-{\overline{C} }_{\mathrm{F},\mathrm{E}\mathrm{C}\mathrm{S}}\right|{V}_{\mathrm{i}}}{{\overline{C} }_{\mathrm{F},\mathrm{E}\mathrm{C}\mathrm{S}}\sum {V}_{\mathrm{i}}}$$

where ${\overline{C} }_{\mathrm{F},\mathrm{E}\mathrm{C}\mathrm{S}}$ is the spatially averaged concentration of free drugs in the tissue extracellular space.

#### Sensitivity Analysis

The sensitivity of each of the above-mentioned qualification indicators to the key properties of brain tissue and drug delivery systems is analysed by

15 $${S}_{\mathrm{i},\mathrm{j}}=\left|\frac{{Y}_{\mathrm{i},\mathrm{j}}-{Y}_{\mathrm{r}\mathrm{e}\mathrm{f}}}{{X}_{\mathrm{i},\mathrm{j}}-{X}_{\mathrm{r}\mathrm{e}\mathrm{f}}}\frac{{X}_{\mathrm{r}\mathrm{e}\mathrm{f}}}{{Y}_{\mathrm{r}\mathrm{e}\mathrm{f}}}\right|$$

where S is the sensitivity. Y is the qualification indicator, and X is the corresponding value of the examined property. The subscripts $\mathrm{i}$, $\mathrm{j}$, and $\mathrm{r}\mathrm{e}\mathrm{f}$ stand for the type of the qualification indicator, the ordinal number of the selected values of the property, and the reference delivery conditions, respectively. Therefore, the sensitivity of each indicator is calculated by

16 $${S}_{\mathrm{i}}=\mathrm{a}\mathrm{v}\mathrm{e}\mathrm{r}\mathrm{a}\mathrm{g}\mathrm{e} ({S}_{\mathrm{i},1, } {S}_{\mathrm{i},2}, \dots , {S}_{\mathrm{i},\mathrm{M}})$$

where M is the total number of selected values of the examined property.

To enable cross-comparison of the impact of each property, the sensitivity of each indicator is further normalised by

17 $$\widetilde{{S}_{\mathrm{i}}}=\frac{{S}_{\mathrm{i}}}{\mathrm{m}\mathrm{a}\mathrm{x}\left({S}_{1}, {S}_{2}, \dots , {S}_{\mathrm{N}}\right)}$$

where N is the total number of examined properties.

#### Characteristic Rate of Drug Transport Mechanisms

The delivery of nanocarriers and the released free drug is governed by multiple mechanisms, as illustrated in Fig. 1. Following the rearrangement of the governing equations, the processes governing nanocarrier and free drug delivery can be decomposed into three components: apparent release, apparent elimination, and apparent transport, as shown in Eqs. (5) and (8), respectively. The apparent transport term incorporates both convective and diffusive mechanisms.

For nanocarriers, the characteristic rates associated with the apparent release and apparent elimination processes are represented by ${k}_{\mathrm{r}\mathrm{e}\mathrm{l},\mathrm{N}}^{*}$ and ${k}_{\mathrm{e}\mathrm{l}\mathrm{m},\mathrm{N}}^{*}$, respectively. The characteristic transport rate is defined as ${k}_{\mathrm{t}\mathrm{r}\mathrm{a}\mathrm{n},\mathrm{N}}^{*}=\frac{{D}_{\mathrm{N},\mathrm{E}\mathrm{C}\mathrm{S}}^{*}}{{l}^{2}}+\frac{{\mathrm{u}}_{\mathrm{N}}^{*}}{l}$, where ${\mathrm{u}}_{\mathrm{N}}^{*}$ is the magnitude of the interstitial fluid flow. The transport length scale, l, is set to be $2 \mathrm{c}\mathrm{m}$, corresponding to the common spatial extent over which tumour recurrence is likely to occur [7].

To quantify the relative importance of apparent release and apparent transport in determining nanocarrier delivery, the apparent release-transport number for nanocarriers, ${\mathrm{C}\mathrm{R}}_{\mathrm{r}\mathrm{e}\mathrm{l}/\mathrm{t}\mathrm{r}\mathrm{a}\mathrm{n},\mathrm{N}}$, is introduced as

18 $${\mathrm{C}\mathrm{R}}_{\mathrm{r}\mathrm{e}\mathrm{l}/\mathrm{t}\mathrm{r}\mathrm{a}\mathrm{n},\mathrm{N}}=\frac{{k}_{\mathrm{r}\mathrm{e}\mathrm{l},\mathrm{N}}^{*}}{{k}_{\mathrm{t}\mathrm{r}\mathrm{a}\mathrm{n},\mathrm{N}}^{*}}$$

The value of ${\mathrm{C}\mathrm{R}}_{\mathrm{r}\mathrm{e}\mathrm{l}/\mathrm{t}\mathrm{r}\mathrm{a}\mathrm{n},\mathrm{N}}>1.0$ indicates that the apparent release process dominates over apparent transport in governing nanocarrier delivery, whereas ${\mathrm{C}\mathrm{R}}_{\mathrm{r}\mathrm{e}\mathrm{l}/\mathrm{t}\mathrm{r}\mathrm{a}\mathrm{n},\mathrm{N}}<1.0$ indicates that apparent transport is the dominant mechanism. ${\mathrm{C}\mathrm{R}}_{\mathrm{r}\mathrm{e}\mathrm{l}/\mathrm{t}\mathrm{r}\mathrm{a}\mathrm{n},\mathrm{N}}=1.0$ shows that apparent release and apparent transport processes have equal contributions.

Similarly, the apparent elimination-transport number for nanocarriers, ${\mathrm{C}\mathrm{R}}_{\mathrm{e}\mathrm{l}\mathrm{m}/\mathrm{t}\mathrm{r}\mathrm{a}\mathrm{n},\mathrm{N}}$, can be defined as

19 $${\mathrm{C}\mathrm{R}}_{\mathrm{e}\mathrm{l}\mathrm{m}/\mathrm{t}\mathrm{r}\mathrm{a}\mathrm{n},\mathrm{N}}=\frac{{k}_{\mathrm{e}\mathrm{l}\mathrm{m},\mathrm{N}}^{*}}{{k}_{\mathrm{t}\mathrm{r}\mathrm{a}\mathrm{n},\mathrm{N}}^{*}}$$

The value of ${\mathrm{C}\mathrm{R}}_{\mathrm{e}\mathrm{l}\mathrm{m}/\mathrm{t}\mathrm{r}\mathrm{a}\mathrm{n},\mathrm{N}}>1.0$ suggests that apparent elimination exerts a stronger influence than apparent transport on nanocarrier delivery, while a value below unity indicates the opposite. ${\mathrm{C}\mathrm{R}}_{\mathrm{e}\mathrm{l}\mathrm{m}/\mathrm{t}\mathrm{r}\mathrm{a}\mathrm{n},\mathrm{N}}=1.0$ indicates that apparent elimination and apparent transport contribute equally to nanocarrier delivery.

Analogous dimensionless numbers can be used to assess the relative importance of the delivery mechanisms governing free drug. The apparent release-transport number for the free drug, ${\mathrm{C}\mathrm{R}}_{\mathrm{r}\mathrm{e}\mathrm{l}/\mathrm{t}\mathrm{r}\mathrm{a}\mathrm{n},\mathrm{F}}$, is given by

20 $${\mathrm{C}\mathrm{R}}_{\mathrm{r}\mathrm{e}\mathrm{l}/\mathrm{t}\mathrm{r}\mathrm{a}\mathrm{n},\mathrm{F}}=\frac{{k}_{\mathrm{r}\mathrm{e}\mathrm{l},\mathrm{F}}^{*}{C}_{\mathrm{E}\mathrm{C}\mathrm{S},\mathrm{N}}}{{k}_{\mathrm{t}\mathrm{r}\mathrm{a}\mathrm{n},\mathrm{F}}^{*}{C}_{\mathrm{E}\mathrm{C}\mathrm{S},\mathrm{F}}}$$

where ${k}_{\mathrm{t}\mathrm{r}\mathrm{a}\mathrm{n},\mathrm{F}}^{*}$ denotes the characteristic rate of apparent free drug transport in the tumour tissue and is defined as ${k}_{\mathrm{t}\mathrm{r}\mathrm{a}\mathrm{n},\mathrm{N}}^{*}=\frac{{D}_{\mathrm{F},\mathrm{E}\mathrm{C}\mathrm{S}}^{*}}{{l}^{2}}+\frac{{\mathrm{u}}_{\mathrm{F}}^{*}}{l}$. It should be noted that the apparent release process is governed by the nanocarrier concentration, whereas apparent transport depends on the concentration of free drug. Therefore, both concentrations are incorporated into Eq. (20). A value of ${\mathrm{C}\mathrm{R}}_{\mathrm{r}\mathrm{e}\mathrm{l}/\mathrm{t}\mathrm{r}\mathrm{a}\mathrm{n},\mathrm{F}}>1.0$ means that apparent release is the dominant mechanism, while a value below unity suggests apparent transport dominance. ${\mathrm{C}\mathrm{R}}_{\mathrm{r}\mathrm{e}\mathrm{l}/\mathrm{t}\mathrm{r}\mathrm{a}\mathrm{n},\mathrm{F}}=1.0$ shows that apparent release and apparent transport in brain tissue are equally important in determining free drug delivery.

The apparent elimination-transport number for the free drug, ${\mathrm{C}\mathrm{R}}_{\mathrm{e}\mathrm{l}\mathrm{m}/\mathrm{t}\mathrm{r}\mathrm{a}\mathrm{n},\mathrm{F}}$, is defined as

21 $${\mathrm{C}\mathrm{R}}_{\mathrm{e}\mathrm{l}\mathrm{m}/\mathrm{t}\mathrm{r}\mathrm{a}\mathrm{n},\mathrm{F}}=\frac{{k}_{\mathrm{e}\mathrm{l}\mathrm{m},\mathrm{F}}^{*}}{{k}_{\mathrm{t}\mathrm{r}\mathrm{a}\mathrm{n},\mathrm{F}}^{*}}$$

A value of ${\mathrm{C}\mathrm{R}}_{\mathrm{e}\mathrm{l}\mathrm{m}/\mathrm{t}\mathrm{r}\mathrm{a}\mathrm{n},\mathrm{F}}>1.0$ indicates that apparent elimination plays a more important role than apparent transport in determining free drug delivery, whereas a value below unity shows that apparent transport is the dominant mechanism. The equal contributions of apparent elimination and apparent transport result in ${\mathrm{C}\mathrm{R}}_{\mathrm{e}\mathrm{l}\mathrm{m}/\mathrm{t}\mathrm{r}\mathrm{a}\mathrm{n},\mathrm{F}}=1.0$.

## Results

### Baseline Study

**Fig. 3:**
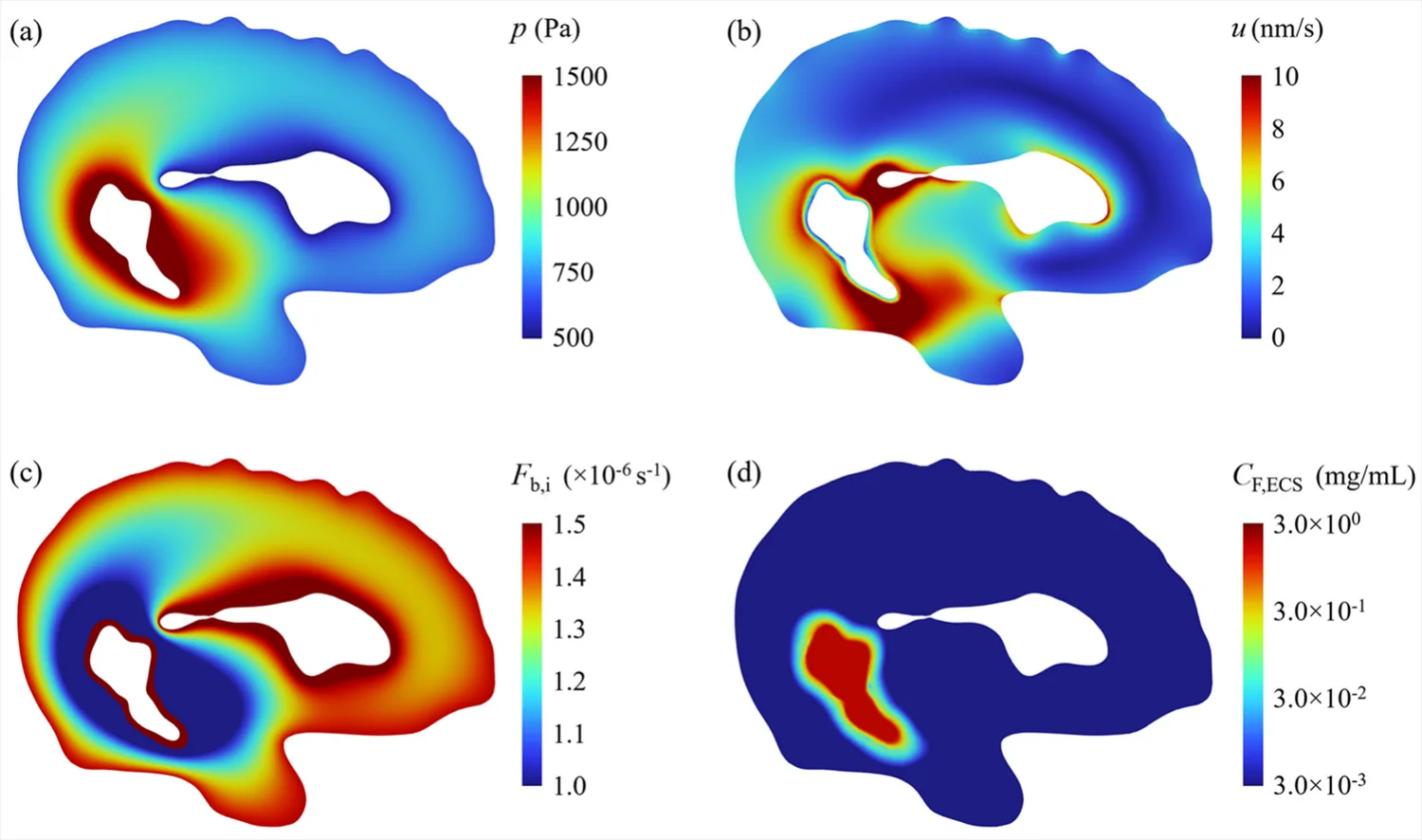
Spatial distribution of interstitial fluid flow and delivered drug. Interstitial fluid pressure (**a**) and velocity (**b**), the plasma leakage rate from the capillaries to the brain tissue (**c**), and the free drug concentration in the extracellular space of the brain tissue (**d**).

The oedema originating from the traumatic region enhances the perfusion of interstitial fluid in the brain parenchyma, within which the drug is conveyed and accumulates. The modelled interstitial fluid flow is presented in Fig. 3. The interstitial pressure is markedly elevated in the brain tissue surrounding the implanted hydrogel, resulting in pronounced pressure gradients across the parenchyma as shown in Fig. 3a. These gradients, in turn, drive accelerated interstitial fluid flow. Figure 3b further indicates that the highest interstitial flow velocities occur in regions adjacent to the ventricular and brain surfaces, where pressure gradients are greatest. However, the velocity of interstitial fluid flow is low within the traumatic region, particularly at the hydrogel surface. In addition, plasma leakage is substantially increased within the traumatic region due to the characteristics of vasogenic oedema. Conversely, the oedema-induced elevation in the interstitial pressure effectively suppresses plasma leakage in the non-traumatic regions surrounding the hydrogel, as illustrated in Fig. 3c.

Figure 3d demonstrates that the implanted hydrogel can successfully deliver drugs into the surrounding brain tissue, although a large proportion of the drug is present within the hydrogel matrix. While drug release into the tissue is evident, localised accumulation indicates that the drug can also diffuse back into the hydrogel. This highlights the hydrogel's role as a sustained drug reservoir, enabling prolonged release and therapeutic action. Further quantitative analysis reveals that drug availability within 2 cm of the pre-operative tumour surface is five orders of magnitude higher than in the regions beyond 2 cm. Given that more than 90% of tumour recurrence occurs within this 2 cm margin [7], the subsequent analysis focuses on drug accumulation in this clinically relevant region adjacent to the hydrogel, which occupies the cavity created by tumour resection.

### Impact of Vascular Hydraulic Conductivity in the Traumatic Region

**Fig. 4:**
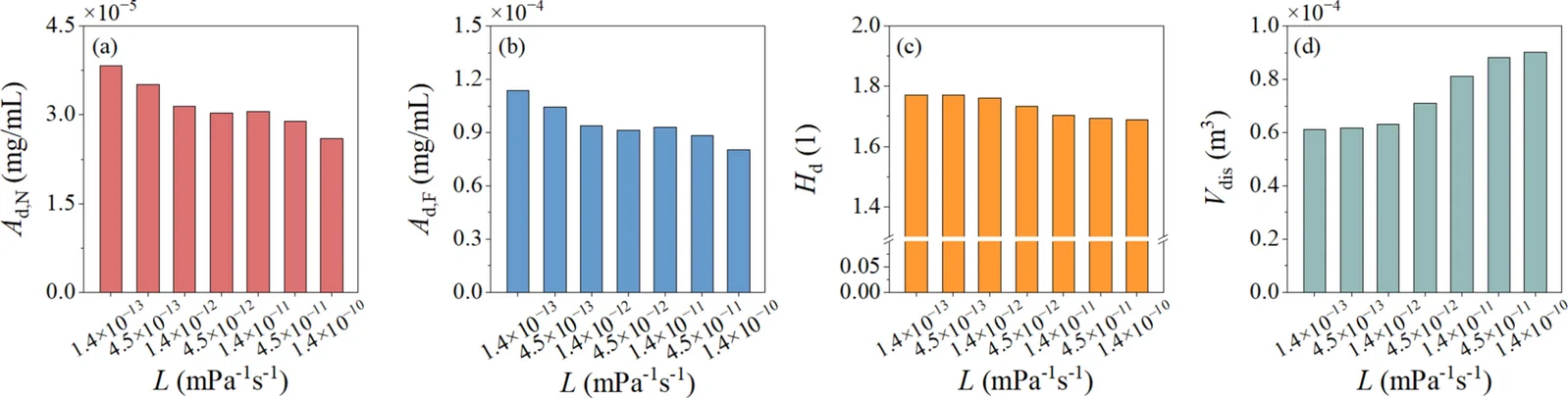
Drug delivery outcomes under the conditions of various capillary wall hydraulic conductivities. (**a**) Availability of nanocarriers, (**b**) availability of free drugs, (**c**) drug distribution non-uniformity, and (**d**) distribution volume.

The influence of the hydraulic conductivity of the capillary wall in the traumatic region on drug delivery outcomes is illustrated in Fig. 4. As shown in Figs. 4a and b, drug availability decreases markedly as the hydraulic conductivity increases from $1.4\times {10}^{-13} \mathrm{m}\hspace{0.17em}{\mathrm{Pa}}^{-1}\hspace{0.17em}{\mathrm{s}}^{-1}$ to $4.5\times {10}^{-12}\hspace{0.17em}\mathrm{m}\hspace{0.17em}{\mathrm{Pa}}^{-1}\hspace{0.17em}{\mathrm{s}}^{-1}$. This reduction arises because enhanced plasma leakage promotes convective transport while, more importantly, diluting the drug concentration within the brain tissue. As a result, a slight decline in distribution non-uniformity and an increase in the distribution volume can be found in Figs. 4c and d, respectively.

With a further increase in hydraulic conductivity, plasma leakage intensifies in the traumatic region, accelerating interstitial fluid flow in the entire brain and facilitating drug transport into deeper tissue. This leads to an expanded distribution volume and a relatively more uniform spatial distribution, see Figs. 4c and d. The enhanced interstitial flow and increased dilution in the traumatic region can reduce local drug availability; however, further mitigated dilution in the non-traumatic region and deeper penetration can contribute to drug accumulation in the non-traumatic region. Therefore, a modest increase in drug availability can be achieved under certain conditions, for example, at $1.4\times {10}^{-11}\hspace{0.17em}\mathrm{m}\hspace{0.17em}{\mathrm{Pa}}^{-1}\hspace{0.17em}{\mathrm{s}}^{-1}$. Moreover, drug availability ultimately decreases with increasing hydraulic conductivity, indicating that the effects of accelerated interstitial flow and enhanced dilution in the traumatic region become dominant as oedema severity increases.

### Impact of Brain Tissue Permeability

**Fig. 5:**
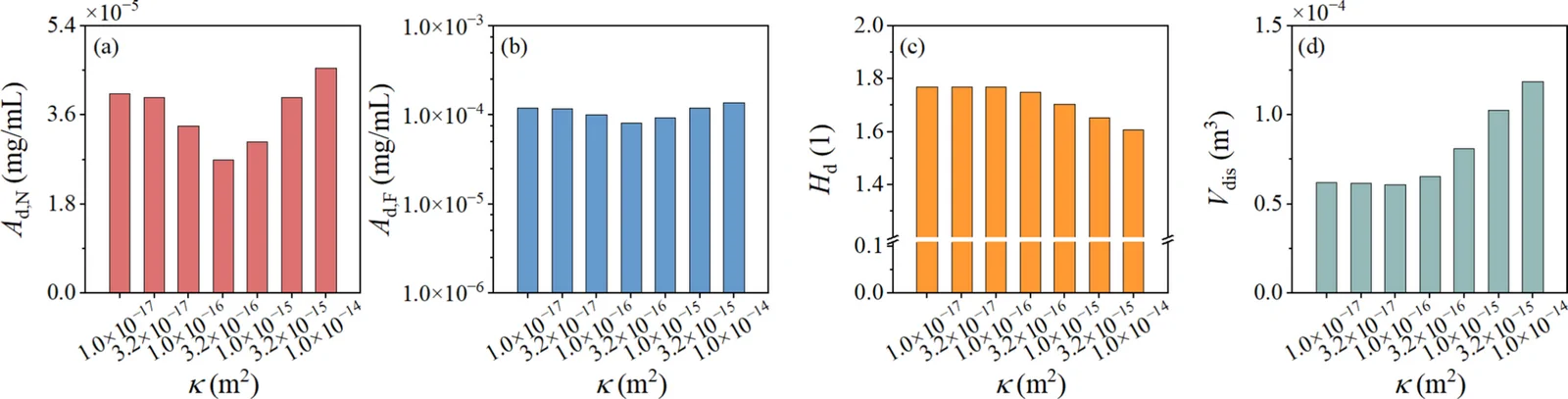
Drug delivery outcomes under the conditions of various tissue permeability. (**a**) Availability of nanocarriers, (**b**) availability of free drugs, (**c**) drug distribution non-uniformity, and (**d**) distribution volume.

Figure 5 illustrates the influence of brain tissue permeability on drug delivery outcomes using an implantable hydrogel. The increase in tissue permeability from $1.0\times {10}^{-17} {\mathrm{m}}^{2}$ to $3.2\times {10}^{-16}\hspace{0.17em}{\mathrm{m}}^{2}$ leads to a reduction in the availability of both nanoparticles and free drug. This may be owing to the elevated interstitial fluid velocities under conditions where the drug distribution non-uniformity and volume remain relatively constant, as shown in Figs. 5c and d.

It is worth noting that, unlike variation in the hydraulic conductivity of capillary walls, which is confined to the traumatic region, changes in tissue permeability occur throughout the brain. Results show that a further increase in tissue permeability continues to expand the distribution volume and homogenises the distribution. This can be attributed to accelerated interstitial fluid flow, which enhances drug convective transport into deeper tissue regions. Such improved penetration may also contribute to the increase in drug availability when the tissue permeability exceeds $3.2\times {10}^{-16}\hspace{0.17em}{\mathrm{m}}^{2}$.

### Impact of Capillary Blood Pressure

**Fig. 6:**
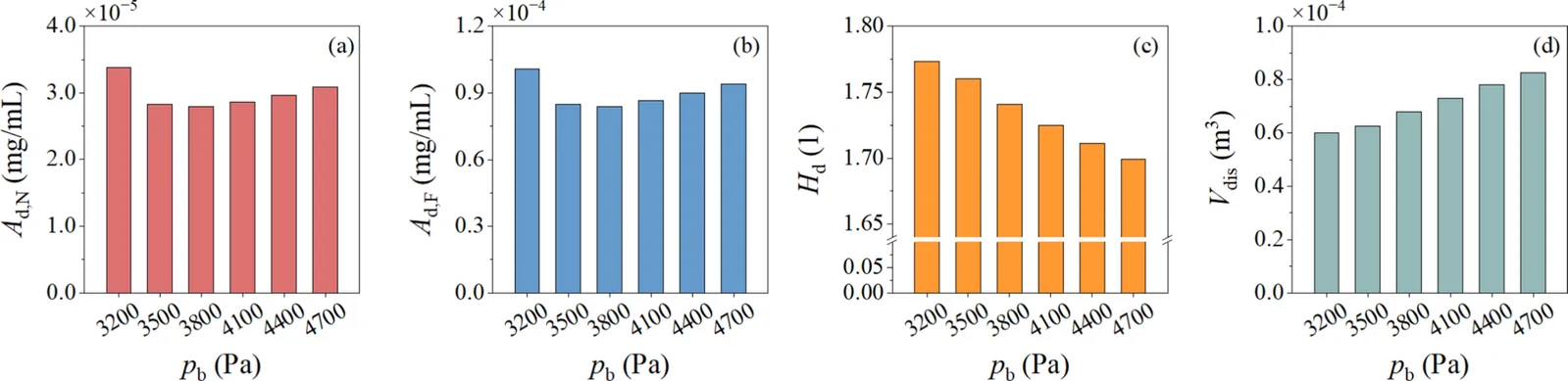
Drug delivery outcomes under the conditions of various capillary blood pressures. (**a**) Availability of nanocarriers, (**b**) availability of free drugs, (**c**) drug distribution non-uniformity, and (**d**) distribution volume.

The effect of capillary blood pressure on drug delivery outcomes is presented in Fig. 6. The results show that drug availability exhibits a non-linear dependence on blood pressure, whereas the distribution volume and distribution non-uniformity vary monotonically with increasing blood pressure. As indicated by Eqs. (1) and (2), the increased blood pressure enhances plasma leakage throughout the brain, thereby accelerating interstitial fluid flow globally. This global acceleration gives rise to two competing effects: it enhances drug washout, thereby reducing drug accumulation, while concurrently increasing the distribution volume, which in turn facilitates more effective drug accumulation. The modelling results indicate that the washout effect dominates when the blood pressure ranges from 3200 to 3500 Pa, leading to reduced drug availability. As blood pressure increases further, the contribution of the enlarged distribution volume becomes dominant, therefore improving drug availability.

### Impact of Drug Release Rate from Nanocarriers

**Fig. 7:**
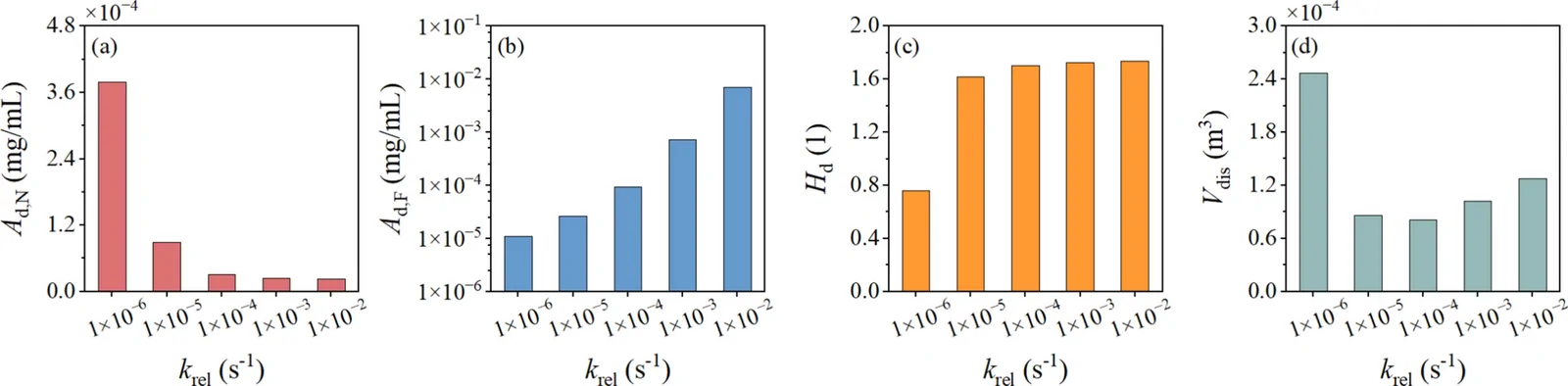
Delivery outcomes using nanocarriers with different drug release rates. (**a**) Availability of nanocarriers, (**b**) availability of free drugs, (**c**) drug distribution non-uniformity, and (**d**) distribution volume.

Figure 7 illustrates the delivery results for nanocarriers with varying drug release rates. Increasing the release rate can improve the conversion of the drug from the nanocarrier-encapsulated form to the free form. Consequently, nanocarrier availability is negatively correlated with the release rate, whereas the availability of free drug shows a positive correlation, as demonstrated in Figs. 7a and b. An interesting observation is that the spatial distribution becomes increasingly non-uniform with higher release rates, see Fig. 7c, primarily due to local accumulation of the released drug.

This behaviour can be explained as follows. At low release rates, a larger fraction of the drug remains encapsulated within nanocarriers, which are less susceptible to elimination via bioreactions and can therefore penetrate deeper into the tissue. As the release rate increases, more of the drug is converted into its free form and is rapidly cleared through blood drainage, physical degradation, and bioreactions. This results in a marked reduction in distribution volume as the release rate increases from $1.0\times {10}^{-6} {\mathrm{s}}^{-1}$ to $1.0\times {10}^{-4} {\mathrm{s}}^{-1}$. However, further increases in the release rate lead to greater drug release, strengthening the concentration gradient of free drug between the hydrogel surface and the brain parenchyma and ventricular regions. Although elimination mechanisms remain active, the enhanced concentration gradient facilitates deeper penetration. As a result, the distribution volume increases again, albeit more gradually than the decline observed at intermediate release rates. The therapeutic distribution volume reaches a minimum at a release rate of $1.0\times {10}^{-4} {\mathrm{s}}^{-1}$. Both lower and higher release rates lead to improved drug penetration.

### Impact of Nanocarrier Diffusivity in the Brain Tissue

**Fig. 8:**
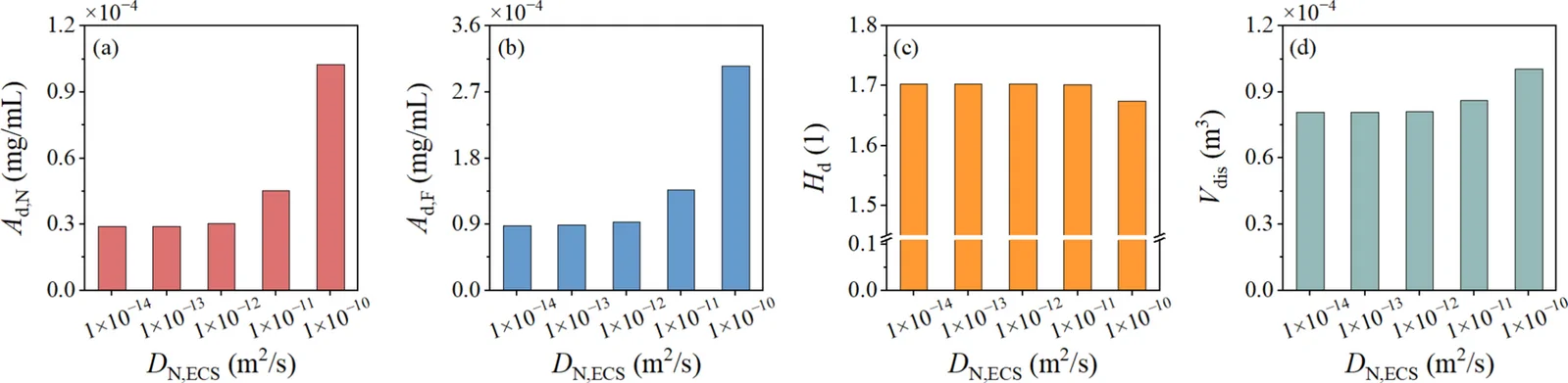
Delivery outcomes under the conditions of different nanocarrier diffusivity. (**a**) Availability of nanocarriers, (**b**) availability of free drugs, (**c**) drug distribution non-uniformity, and (**d**) distribution volume.

Figure 8 presents the delivery results for nanocarriers with different diffusion coefficients in brain tissue. Increasing the diffusion coefficient enhances nanocarrier mobility, thereby strengthening their diffusive flux away from the hydrogel-tissue interface and promoting deeper penetration into the brain parenchyma. This reduces local retention near the source and enables a greater fraction of nanocarriers to be distributed throughout the tissue domain. As a result, the total accumulation of nanocarrier-encapsulated drug increases, since a larger number of nanocarriers are transported into regions that would otherwise remain poorly reached. The elevated nanocarrier presence subsequently leads to increased generation of free drug through sustained release along the transport pathways.

In addition, the spatially distributed release of free drug reduces concentration localisation near the hydrogel and broadens the region over which the drug is supplied. This mechanism effectively smooths concentration gradients, resulting in a more homogeneous free drug distribution. The combined effects of enhanced nanocarrier transport and spatially distributed release ultimately expand the therapeutic coverage volume.

### Impact of Drug Dose Loaded in the Hydrogel

**Fig. 9:**
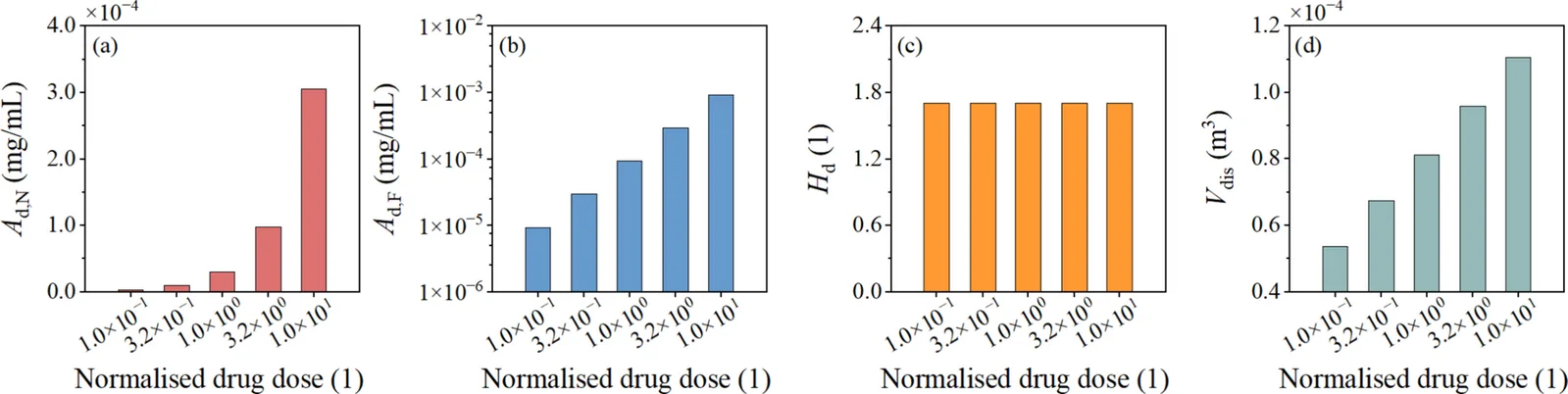
Delivery outcomes under the conditions of different doses. (**a**) Availability of nanocarriers, (**b**) availability of free drugs, (**c**) drug distribution non-uniformity, and (**d**) distribution volume. The dose on the x-axis is normalised by the baseline dose.

The drug dose is a clinically controllable parameter. Its impact is examined by varying the baseline dose by one order of magnitude. The results, shown in Fig. 9, indicate that increasing the dose effectively enhances drug availability in both nanocarrier-encapsulated and free forms, as well as the distribution volume. Mechanistically, a higher dose elevates the overall concentration level, thereby increasing both the amount of drug retained in nanocarriers and the amount released into the free phase. This leads to a stronger concentration field throughout the tissue and, consequently, a larger region exceeding the therapeutic threshold. However, the spatial non-uniformity of the drug distribution remains largely unaffected. This is because the dose primarily scales the magnitude of the concentration field without altering the underlying transport and reaction mechanisms that govern the distribution pattern.

### Impact of Nanocarrier Apparent Partition Between Intra- and Extra-cellular Space

**Fig. 10:**
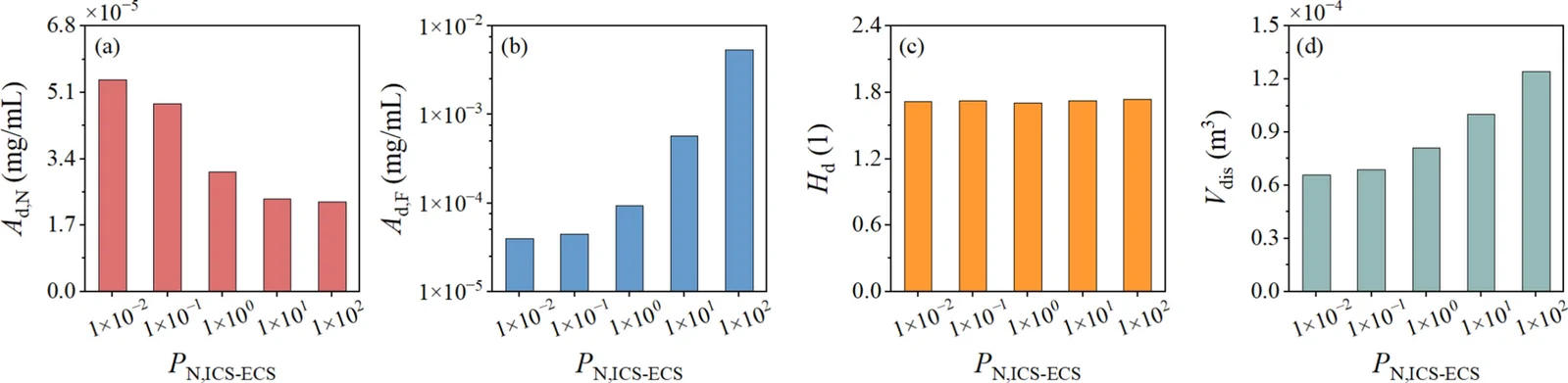
Delivery outcomes under the conditions of different nanocarrier apparent partition coefficients between the intracellular and extracellular space. (**a**) Availability of nanocarriers, (**b**) availability of free drugs, (**c**) drug distribution non-uniformity, and (**d**) distribution volume.

Figure 10 presents the effects of nanocarriers with different apparent partition coefficients between the intracellular and extracellular spaces. Increasing this partition coefficient reduces nanocarrier availability in the extracellular space, as a larger fraction of nanocarriers preferentially partitions into the intracellular compartment. Notably, the increased intracellular nanocarrier accumulation continues to contribute to free drug generation through sustained release within cells. Owing to the establishment of a dynamic equilibrium between the intracellular and extracellular free drug concentrations, the released drug can redistribute across the cell membrane, thereby elevating the free drug availability in the extracellular space. This exchange mechanism effectively enhances overall drug distribution and expands the region of therapeutic relevance. However, the spatial distribution pattern of the drug remains largely unchanged. This is because the apparent partition coefficient primarily redistributes the drug between compartments without altering the governing transport pathways or spatial gradients that define the distribution profile.

### Impact of Nanocarrier Apparent Partition Between Cell Membrane and Extracellular Space

**Fig. 11:**
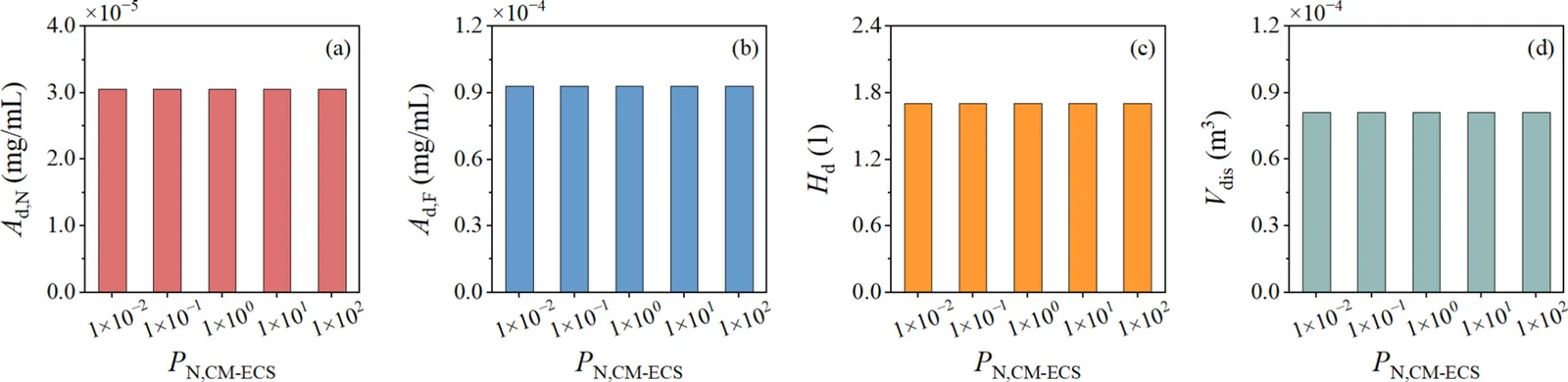
Delivery outcomes under the conditions of different nanocarrier partition coefficients between the cell membrane and the extracellular space. (**a**) Availability of nanocarriers, (**b**) availability of free drugs, (**c**) drug distribution non-uniformity, and (**d**) distribution volume.

Figure 11 compares the performances of nanocarriers with different apparent partition coefficients between the cell membrane and extracellular space. The modelling results indicate that drug availability, spatial distribution patterns, and distribution volume are largely insensitive to this parameter. The partitioning at the cell membrane does not introduce a net sink or source for nanocarriers, as there is no associated elimination or permanent deposition at the membrane. Instead, the membrane acts as a transient interface through which nanocarriers can reversibly associate and dissociate. As a result, changes in the apparent partition coefficient only redistribute nanocarriers locally at the interface without altering their overall transport pathways or residence time within the tissue. Consequently, the global drug transport and release dynamics remain essentially unchanged when the dynamic equilibrium is established, leading to a negligible impact on the resulting delivery outcomes.

### Impact of Nanocarrier Blood Drainage Rate

**Fig. 12:**
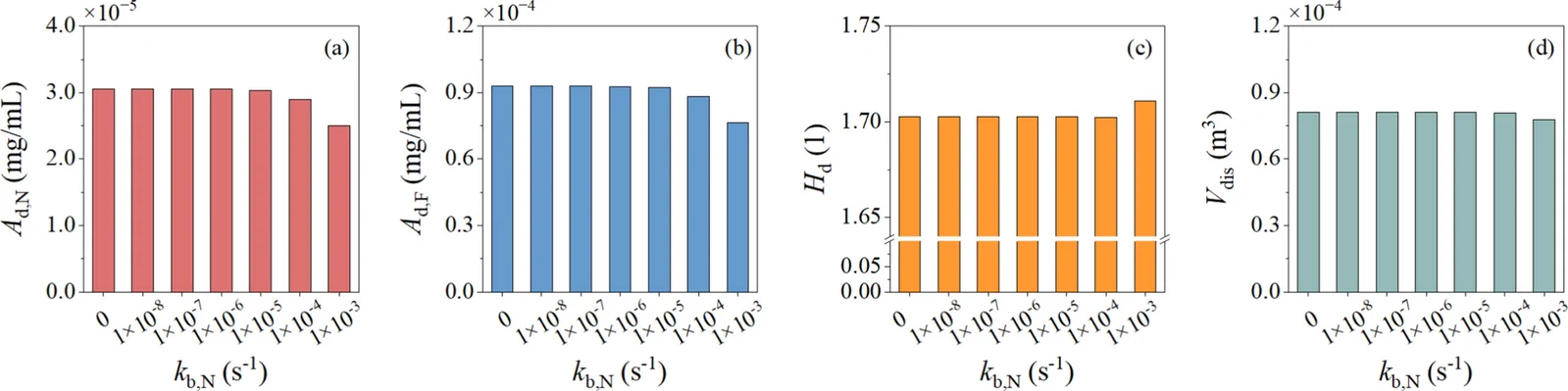
Delivery outcomes under the conditions of different nanocarrier blood drainage rates. (**a**) Availability of nanocarriers, (**b**) availability of free drugs, (**c**) drug distribution non-uniformity, and (**d**) distribution volume.

Figure 12 summarises the delivery outcomes for nanocarriers with different transfer rates into the blood capillaries. Acting as a sink, capillary uptake enhances the clearance of nanocarriers from the brain tissue. As the transfer rate increases, nanocarrier removal from the extracellular space becomes more efficient, leading to a reduction in nanocarrier availability within the tissue. This diminished nanocarrier presence subsequently reduces the deposition of free drug, as fewer nanocarriers remain available for sustained release. In addition, the enhanced clearance lowers the overall concentration level, thereby weakening the concentration gradients that drive drug penetration into deeper regions. Consequently, the distribution volume decreases, and the spatial distribution of free drug becomes more heterogeneous. This is primarily due to the limited penetration depth, with drug concentrations becoming increasingly confined to regions proximal to the source.

### Sensitivity Analysis

**Fig. 13:**
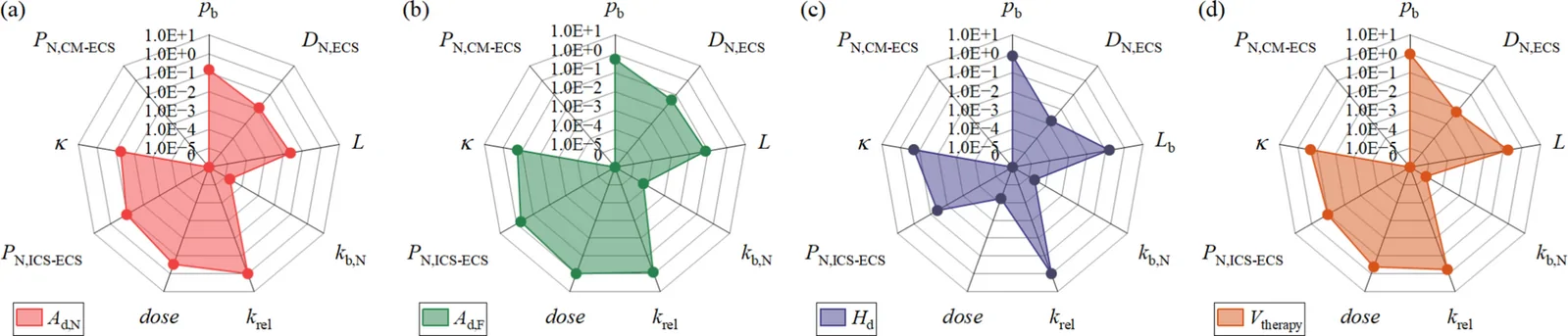
Sensitivity analysis of the delivery outcomes to each influential factor. The sensitivity, $\widetilde{{S}_{\mathrm{i}}}$, of availability of (**a**) nanocarriers and (**b**) free drug, (**c**) distribution non-uniformity, and (**d**) distribution volume.

Figure 13 presents a ranking of the relative importance of key tissue and delivery parameters, using the baseline study as the reference condition. Under oedematous conditions, nanocarrier availability is primarily governed by the release rate, followed by drug dose, blood pressure, nanocarrier apparent intracellular-extracellular space partitioning, tissue permeability, and the hydraulic conductivity of the vascular wall. Secondary influences include nanocarrier diffusivity, blood drainage, and nanocarrier apparent cell membrane-extracellular space partitioning. In contrast, free drug availability exhibits a different dependency pattern. While the least five influential factors remain unchanged, free drug levels are more strongly determined by drug dose, release rate, nanocarrier intracellular-extracellular space partitioning, and blood pressure.

Importantly, trends in drug availability do not directly translate to distribution volume. The latter is mainly controlled by blood pressure and release kinetics, followed by drug dose loaded in the hydrogel, tissue permeability, vascular hydraulic conductivity, and the nanocarrier’s apparent intracellular-extracellular space partitioning. Other factors, including nanocarrier diffusivity, blood drainage, and apparent cell membrane-extracellular space partitioning of nanocarriers, play comparatively minor roles.

Drug distribution non-uniformity shows yet another distinct pattern, being mainly controlled by release rate and blood pressure, with tissue permeability, hydraulic conductivity, apparent intracellular-extracellular space partitioning and diffusivity playing a secondary role of nanocarriers. Whereas drug dose, blood drainage, and the nanocarrier’s apparent cell membrane-extracellular space partitioning exert relatively limited influence.

### Relative Importance of Delivery Mechanisms

**Fig. 14:**
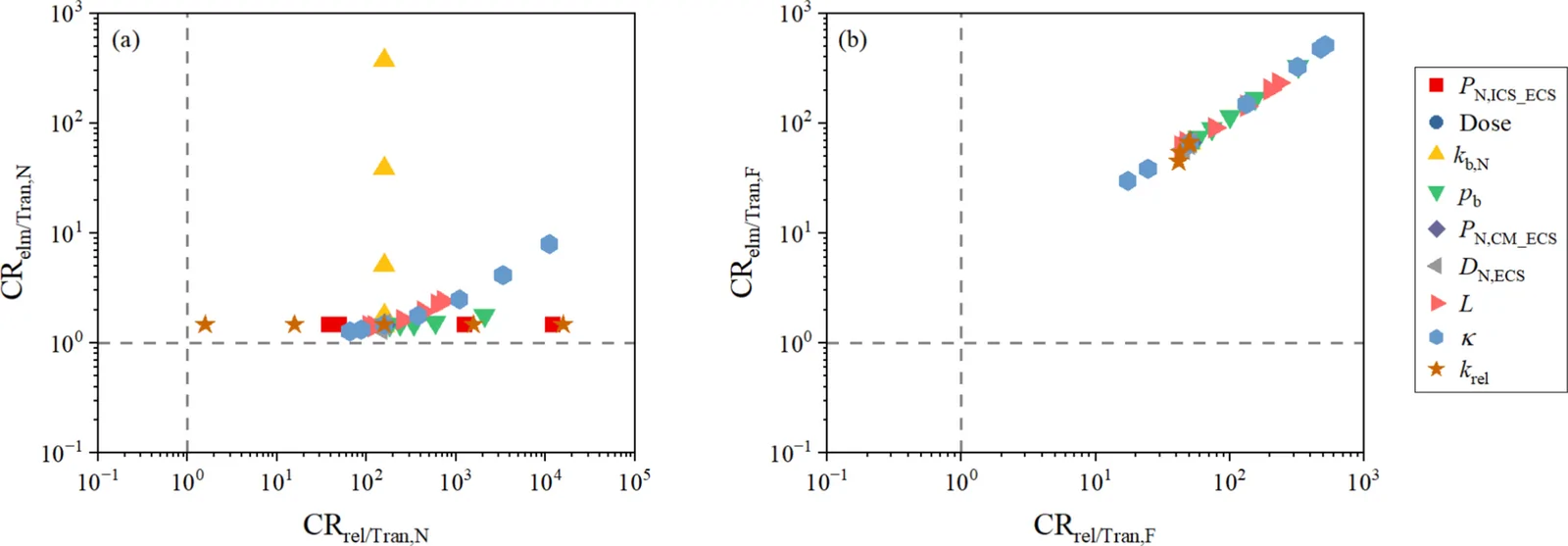
Regime map of delivery mechanisms for nanocarriers (**a**) and free drug (**b**).

Figure 14 compares the relative contributions of different delivery mechanisms in determining the delivery outcomes of nanocarriers and free drug. The regime maps are divided into four regions by the lines corresponding to the apparent release-transport number and the apparent elimination-transport number of unity.

For nanocarriers shown in Fig. 14a, all investigated cases are located in the upper-right region of the regime map. This indicates that both apparent release and apparent elimination play more important roles than apparent transport in determining nanocarrier delivery. Further comparison across different nanocarrier and tissue properties reveals that variations in blood drainage predominantly affect the importance of apparent elimination, while having a negligible influence on the relative contribution of apparent release. In contrast, changes in the tissue permeability effectively alter the relative importance of both apparent release and apparent elimination. This effect is followed by changes in the hydraulic conductivity of the capillary wall in the traumatic region and blood pressure in sequence; they are all properties of the brain tissue. Variations in the remaining parameters mainly influence the relative contribution of apparent release, while exerting only a limited effect on apparent elimination. Moreover, except for blood drainage rate, variations in all other parameters produce a much wider range of changes in the relative contribution of apparent release than in that of apparent elimination. This finding suggests that the relative influence of the apparent release mechanism on nanocarrier delivery is more sensitive to most nanocarrier properties and tissue characteristics than the apparent elimination mechanism.

**Fig. 15:**
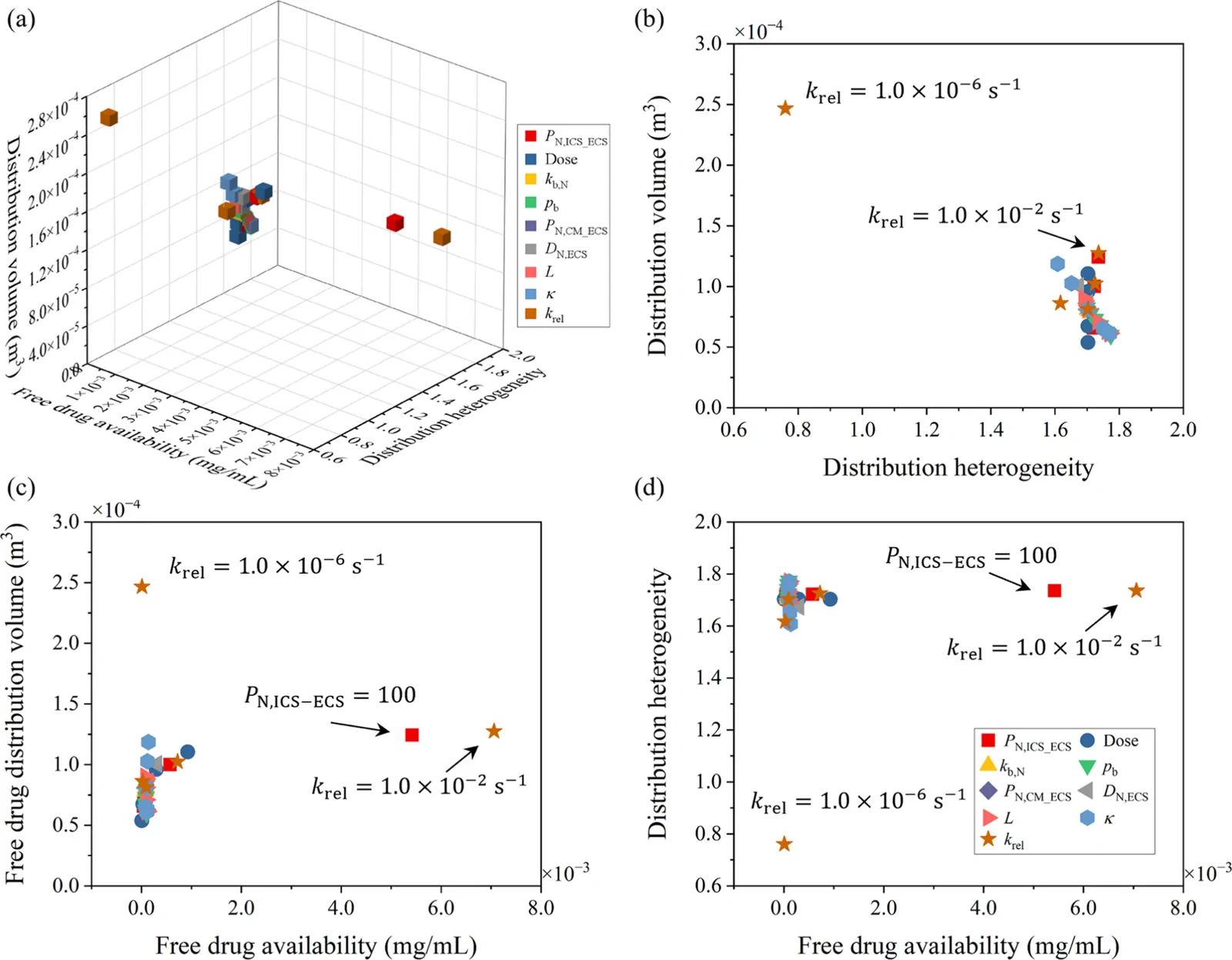
Cross-comparison of delivery outcomes under various delivery conditions

The delivery outcomes of free drug shown in Fig. 15b are also largely governed by apparent release and apparent elimination. Across all investigated nanocarrier and tissue properties, these two mechanisms exhibit comparable levels of importance. Notably, variations in the tissue permeability lead to the largest changes in their relative contributions.

## Discussion

In the oedematous post-operative brain, interstitial pressure is significantly higher in the vicinity of the implanted hydrogel filling the resection cavity, primarily due to enhanced plasma leakage in trauma-affected tissue. This establishes a local pressure gradient that drives elevated interstitial fluid flow while restricting plasma leakage in non-traumatised regions. The former is expected to enhance convective transport, thereby reducing local drug concentration but promoting deeper penetration, whereas the latter may mitigate dilution and sustain higher local drug levels. Simulation results confirm that the implanted hydrogel enables effective drug delivery into the surrounding brain tissue, producing a monotonic concentration gradient that decays from the hydrogel interface into the parenchyma.

The parametric analysis indicates that delivery outcomes can be enhanced by strategically tuning the coupled mechanisms of transport kinetics, partitioning behaviour, and tissue properties. Increasing drug loading within the hydrogel represents a direct and effective approach, as it elevates the concentration gradient at the deployment interface, thereby strengthening diffusive flux, increasing drug availability, and extending spatial coverage. Similarly, moderately increasing the nanocarrier release rate can further improve free drug availability in the extracellular space, but leads to more heterogeneous distributions; this strategy must also be carefully controlled, since relatively rapid release (approaching $O(-4)\;s-^1$) can cause local depletion and insufficient penetration time, ultimately limiting the distribution volume. Enhancing nanocarrier internalisation capacity provides another viable route to improve delivery efficiency. Although higher apparent nanocarrier partition coefficients between the intracellular and extracellular space promote accumulation, they also sustain the transmembrane exchange of free drug, which indirectly expands drug distribution without influencing the distribution non-uniformity. Increasing nanocarrier diffusivity, such as through reducing particle size or modifying surface charge, can facilitate interstitial transport for higher drug availability, reduce concentration heterogeneity, and promote deeper tissue penetration. Nanocarriers that can penetrate the blood vessel wall are able to reduce the overall delivery outcomes. Therefore, unlike drug delivery through intravenous administration, nanocarriers that are impermeable to the BBB are favoured in this hydrogel-mediated delivery. In addition to nanocarrier parameters, modulating tissue-level conditions can further influence delivery outcomes. For example, increasing blood pressure enhances vascular filtration and interstitial fluid velocity, strengthening convective transport and improving spatial coverage and uniformity, although this may come at the cost of increased drug washout and reduced local availability. In oedematous tissue, altered hydraulic conductivity can be leveraged to promote long-range transport and deeper penetration, despite reduced local accumulation. Optimal tissue permeability exists for achieving better drug accumulation and enlarged, relatively uniform distribution. Overall, these findings suggest that improving drug delivery effectiveness relies on coordinated optimisation strategies that enhance transport processes while carefully managing clearance and sequestration effects, thereby maximising therapeutic benefit.

Based on the modelling results, Table III summarises potential strategies for tuning the nanocarrier-hydrogel delivery system to enhance delivery outcomes. It is also worth noting that different system properties may require opposing design strategies. For example, increasing nanocarrier diffusivity and intracellular partitioning may favour a reduction in nanocarrier size, whereas decreasing nanocarrier loss to the blood may require an increase in nanocarrier dimensions. In such cases, alternative design strategies can be explored to modify these delivery system parameters to improve overall delivery performance, or priority can be given to delivery system parameters to which the delivery outcomes are more sensitive. However, the latter approach may still partially offset the overall gains in delivery efficiency. Therefore, system-specific analysis is recommended to optimise the design of individual hydrogel-nanocarrier delivery systems. In addition, co-delivery of agents that modulate tissue properties, such as blood pressure, may further improve delivery outcomes. However, such interventions may also induce more complex and potentially unintended physiological responses. The implications of these effects should therefore be carefully evaluated prior to implementation.

**Table III:** Possible Design Strategies for Improved Delivery Outcomes

Delivery system property	Recommendation	Possible design strategy	Sources
Nanocarrier diffusivity	Increase	• Increase nanocarrier surface charge • Reduce nanocarrier size	[72, 73]
Nanocarrier blood drainage rate	Decrease	• Increase nanocarrier size • Remove BBB-targeting ligands • Reduce nanocarrier hydrophobicity	[74, 75]
Drug release rate	Optimise	• Modify carrier degradation rate • Alter drug-carrier interactions	[76, 77]
Nanocarrier loading in hydrogel	Increase	• Increase nanocarrier-hydrogel affinity • Modify nanocarrier surface chemistry • Introduce nanocarrier-hydrogel electrostatic interactions	[78, 79]
Nanocarrier ICS-ECS partition	Increase	• Reduce nanocarrier size • Increase positive surface charge • Introduce cell-targeting ligands • Enhance nanocarrier-cell membrane interactions	[80, 81]

Figure 15a compares the delivery outcomes obtained under different conditions in terms of drug availability, distribution volume, and distribution heterogeneity. The projections of these results onto each pair of delivery metrics are further presented in Figs. 15b–d. The results show that, for most investigated conditions, the delivery outcomes are relatively close. However, increasing ${P}_{\mathrm{N},\mathrm{I}\mathrm{C}\mathrm{S}-\mathrm{E}\mathrm{C}\mathrm{S}}$ substantially enhances free-drug availability and leads to a modest increase in distribution volume, while exerting little influence on distribution heterogeneity. In contrast, variations in the release rate produce much more pronounced effects on the delivery outcomes. Specifically, slowly releasing nanocarriers achieve the largest distribution volume and the most homogeneous drug distribution, although they provide only limited improvement in free-drug availability. Conversely, rapidly releasing nanocarriers result in the highest free-drug availability but yield only marginal improvements in distribution volume and distribution heterogeneity. These findings suggest that optimisation of nanocarrier properties can be used to improve either drug availability or drug distribution within the tissue. However, simultaneous enhancement of both availability and distribution remains challenging, as improvements in one aspect are often accompanied by compromises in the other.

**Fig. 16:**
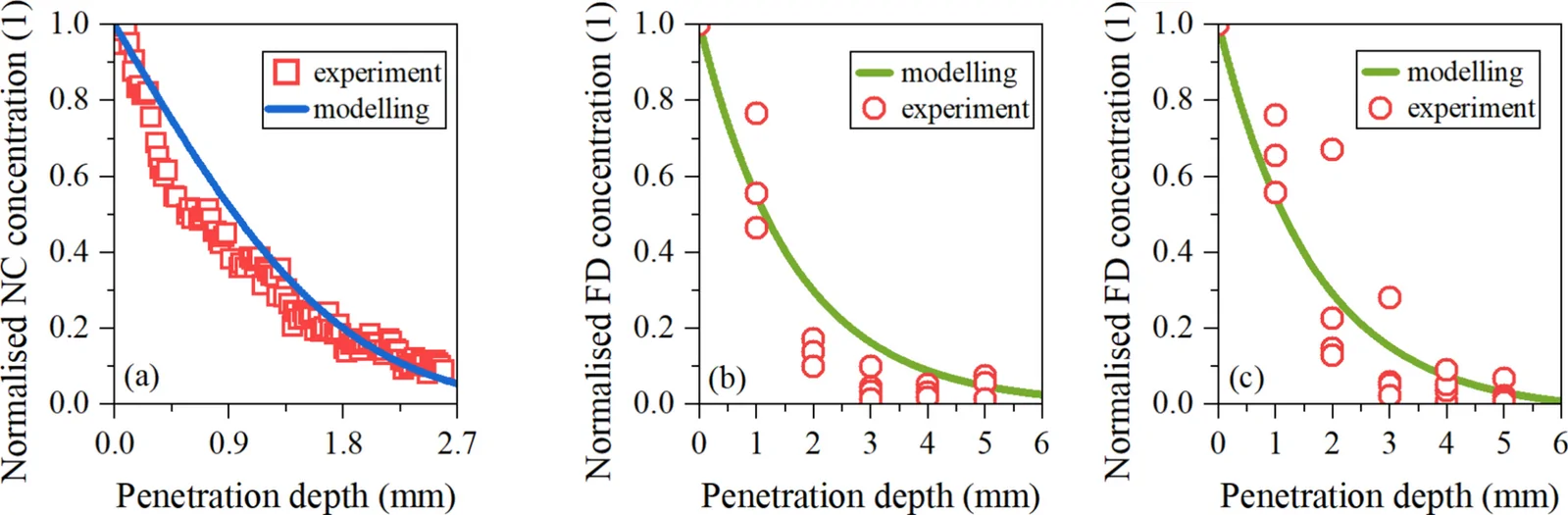
Comparison of simulation results with experimental measurements. Nanocarrier concentration as a function of penetration depth at 12 h after the initiation of delivery (**a**). Paclitaxel concentration as a function of penetration depth at 14 days (**b**) and 28 days (**c**). The drug properties and experimental data for nanocarriers and paclitaxel are obtained from the literature [50] and [82], respectively.

The model is designed to capture the key transport processes governing drug delivery from the hydrogel to the surrounding brain tissue. Modelling predictions show good agreement with experimental measurements [50, 82] in Fig. 16, supporting its predictive capability. It should be noted that the model validation studies are subject to several practical constraints. On the one hand, treatment efficacy is typically assessed using macroscopic metrics such as changes in tumour volume or survival, rather than detailed spatiotemporal distributions of drug concentration in most experimental studies [10, 13]. However, due to the lack of information on the density and spatial distribution of residual tumour cells derived from the available medical imaging data, tumour cell-related metrics are not included in this study. On the other hand, experimental studies generally report treatment outcomes, whereas the tissue-specific properties required for mechanistic simulations, such as those listed in Table I, are rarely available. As a result, it is difficult to accurately reproduce the tissue microenvironment of the experimental system, despite its important influence on drug transport and therapeutic outcomes. Nevertheless, the governing equations for nanocarrier transport are formulated based on the principle of mass conservation, which is consistent with the framework used for free drug transport. In addition, the nanocarrier transport model has been previously compared with* in vivo* experimental data for an alternative drug delivery strategy in our earlier study [83]. Therefore, the underlying transport framework remains consistent across these applications, and the validation studies in Fig. 16 still provide confidence in the correctness of the core transport physics.

Several assumptions and limitations should be acknowledged.

1. The present framework assumes a quasi-steady-state condition, under which drug transport is considered to have reached a dynamic equilibrium. This assumption is motivated by the disparity in timescales between hydrogel-mediated release and oedema (typically days and weeks) and tissue-level transport processes (minutes to hours); the latter can be estimated using $R/\sqrt{{D}_{\mathrm{F},\mathrm{E}\mathrm{C}\mathrm{S}}/{k}_{\mathrm{e}\mathrm{l}\mathrm{m},\mathrm{F}}}$, where R is the equivalent radius of the brain. Similar approximations have been adopted in previous studies on drug delivery to the brain [25]. This equilibrium implies a balance between diffusion, convection, elimination, and cellular exchange processes. However, both oedema progression and drug transport are inherently dynamic. Transient modelling would therefore enable resolution of temporal concentration evolution and capture time-dependent coupling between interstitial fluid dynamics and transport. All the steady-state simulations in this study are conducted on a workstation equipped with 36 Intel® Xeon® W-2295 CPU cores (3.00 GHz). The average computational time for each simulation is approximately four hours. Consequently, performing transient simulations would substantially increase the computational cost and simulation time, particularly considering the large number of parametric studies undertaken in this work. Although simplified piecewise approaches have been reported [41], a comprehensive mechanistic description of oedema evolution remains lacking, which limits the incorporation of fully time-dependent behaviour in the current study.
2. Brain tissue anisotropy, arising from aligned axonal structures, is not considered. This anisotropy can influence directional diffusivity and convective transport pathways. Its exclusion is primarily due to the limited availability of subject-specific data. Future studies could incorporate anisotropic transport tensors derived from diffusion tensor imaging [84] to better resolve direction-dependent transport.
3. The delivery outcomes are evaluated using metrics derived directly from the predicted drug concentrations in this study. It should be noted that these results can be further utilised as inputs to pharmacodynamic models [85] to predict tumour cell killing and simulate changes in tumour cell density. Such analyses are not included in the present study because information regarding the spatial distribution and density of residual tumour cells is not available from the medical imaging data employed. However, the proposed framework could be coupled with appropriate pharmacodynamic models and tumour recurrence-related metrics to provide a more comprehensive evaluation of therapeutic efficacy when such information becomes available in future. Moreover, as the delivered anticancer drugs are transported throughout the post-operative brain tissue, assessment of their effects on normal brain cells is also of considerable importance. The incorporation of suitable models to evaluate both therapeutic efficacy and potential toxicity would provide further insight into treatment outcomes. These investigations would be particularly valuable for the optimisation and personalised design of hydrogel-based drug delivery systems.
4. The effects of multiple tissue and nanocarrier properties are systematically investigated in this study. Parameter values are collected from the literature to span the reported physiological and experimental ranges of each property. It is noted that these values are extracted from different sources under varying experimental conditions; therefore, they should be regarded as representative rather than drug delivery system-specific measurements. Accordingly, this work should be interpreted as an exploratory study aimed at capturing general trends in drug delivery behaviour, rather than being tailored to a specific drug delivery system.
5. This study focuses on the effects of individual tissue and nanocarrier properties without explicitly considering their interactions. In practice, multiple factors may vary simultaneously, potentially leading to more complex responses in the delivery outcomes. A fully combined parametric study could therefore be conducted in future work to investigate multi-factor sensitivity, thereby elucidating parameter interactions and potential synergistic effects. Ultimately, such studies would contribute to a more comprehensive understanding of the factors governing drug delivery performance and facilitate the optimisation and design of hydrogel-based drug delivery systems for post-operative brain tumour treatment. However, it is worth noting that such a comprehensive analysis would require substantial computational resources and data storage owing to the rapid growth in the number of simulation scenarios. Therefore, focusing on the most influential parameters identified in the current study may provide a more computationally efficient strategy for future investigations, while still capturing the dominant factors and their interactions that govern drug delivery outcomes. In addition, fractional factorial designs could be employed to reduce the number of required simulations while retaining the ability to evaluate key parameter interactions. Machine learning surrogate models coupled with SHAP (SHapley Additive exPlanations) analysis may further improve computational efficiency by identifying influential parameters and quantifying their contributions to delivery outcomes.
6. The primary focus of the present study is the transport of nanocarriers and released drugs in the hydrogel-mediated nanocarrier delivery system. Therefore, the model assumes that the hydrogel has already been successfully implanted and occupies the target post-operative cavity at the start of treatment. As a result, the implantation and deployment processes themselves are not explicitly considered in the current model. However, in clinical practice, hydrogel administration involves a number of complex processes that may significantly influence delivery outcomes. These include the placement strategy, injection or implantation procedure, hydrogel rheological behaviour during deployment, gelation kinetics, and the extent of cavity coverage achieved following surgery. In particular, unlike the idealised assumption adopted in the present study, complete coverage of the resection cavity may not always be achieved in practice. Variations in cavity coverage could alter the effective contact area between the hydrogel and surrounding tissue, thereby affecting drug release, transport pathways, and ultimately treatment efficacy. In addition, the implantation location and spatial distribution of the hydrogel may vary substantially among patients because of differences in post-operative cavity geometry, tumour location, surgical constraints, and clinical deployment strategies. These factors may influence the distance that drugs must travel to reach infiltrating tumour cells and therefore affect local drug concentrations in target regions. Future studies could address these clinically relevant factors by developing corresponding computational models that explicitly simulate hydrogel deployment, spreading, and gelation within patient-specific post-operative cavities. Coupling such hydrogel implantation models with the transport framework in the present study would enable a systematic investigation of how cavity coverage, hydrogel placement strategy, implantation location, and other practical deployment factors influence treatment outcomes. Such an approach may provide valuable guidance for both hydrogel design and clinical implementation strategies in local brain drug delivery.

## Conclusions

Hydrogel-mediated delivery of nanocarrier-encapsulated drugs to the oedematous post-operative brain is modelled under various delivery conditions. The results confirm the effectiveness of this combined system for anticancer drug delivery and, importantly, elucidate the responses of drug availability and distribution to key properties of brain tissue and nanocarriers. Increasing the drug release rate enhances free drug availability but leads to more heterogeneous distribution, with an optimal rate maximising distribution volume. Enhanced nanocarrier internalisation improves drug availability and expands distribution volume, with negligible impact on distribution heterogeneity; a similar trend is observed for increased drug loading. In contrast, higher nanocarrier blood drainage reduces overall delivery efficiency, favouring non-vascularly penetrable nanocarriers, whereas greater nanocarrier diffusivity improves delivery outcomes. More severe oedema increases distribution volume but may reduce drug availability. Elevated blood pressure or more permeable tissue promotes deeper and more uniform drug penetration, although with a potential reduction in availability. Nanocarrier partitioning at the cell membrane has a negligible effect on overall outcomes. Furthermore, sensitivity analysis indicates that free drug availability and distribution volume are most influenced by drug loading and blood pressure, respectively, while distribution heterogeneity is primarily governed by the release rate. The results indicate that delivery outcomes are governed primarily by drug release and elimination rather than transport mechanisms. These findings guide optimising hydrogel-nanocarrier delivery systems to improve therapeutic efficacy and suppress glioblastoma recurrence in the post-operative brain.

## Data Availability

The datasets generated and/or analysed during the current study are available from the corresponding author upon reasonable request.
